# Pre-deployment risk factors for PTSD in active-duty personnel deployed to Afghanistan: a machine-learning approach for analyzing multivariate predictors

**DOI:** 10.1038/s41380-020-0789-2

**Published:** 2020-06-02

**Authors:** Katharina Schultebraucks, Meng Qian, Duna Abu-Amara, Kelsey Dean, Eugene Laska, Carole Siegel, Aarti Gautam, Guia Guffanti, Rasha Hammamieh, Burook Misganaw, Synthia H. Mellon, Owen M. Wolkowitz, Esther M. Blessing, Amit Etkin, Kerry J. Ressler, Francis J. Doyle, Marti Jett, Charles R. Marmar

**Affiliations:** 1 Department of Psychiatry, New York University Grossman School of Medicine, New York, NY USA; 2 Department of Emergency Medicine, Vagelos School of Physicians and Surgeons, Columbia University Medical Center, New York, NY USA; 3 Data Science Institute, Columbia University, New York, NY USA; 4 Department of Psychiatry, Center for Alcohol Use Disorder and PTSD, New York University Grossman School of Medicine, New York, NY USA; 5 Harvard Paulson School of Engineering & Applied Sciences, Boston, MA USA; 6 Department of Population Health, Biostatistics Division, New York University Grossman School of Medicine, New York, NY USA; 7 Integrative Systems Biology, US Army Center for Environmental Health Research, USACEHR, Fort Detrick, Frederick, MD USA; 8 McLean Hospital, Harvard University, Boston, MA USA; 9 Department of Psychiatry and Behavioral Sciences, Emory University School of Medicine, Atlanta, GA USA; 10 Department of Obstetrics, Gynecology & Reproductive Sciences, University of California, San Francisco, CA USA; 11 Department of Psychiatry/Weill Institute for Neurosciences, University of California, San Francisco, CA USA; 12 Alto Neuroscience, Inc., Los Altos, CA USA; 13 Department of Psychiatry and Behavioral Sciences, Stanford University, Stanford, CA USA; 14 Wu Tsai Neurosciences Institute, Stanford University, Stanford, CA USA

**Keywords:** Genetics, Predictive markers, Psychiatric disorders, Molecular biology, Prognostic markers

## Abstract

Active-duty Army personnel can be exposed to traumatic warzone events and are at increased risk for developing post-traumatic stress disorder (PTSD) compared with the general population. PTSD is associated with high individual and societal costs, but identification of predictive markers to determine deployment readiness and risk mitigation strategies is not well understood. This prospective longitudinal naturalistic cohort study—the Fort Campbell Cohort study—examined the value of using a large multidimensional dataset collected from soldiers prior to deployment to Afghanistan for predicting post-deployment PTSD status. The dataset consisted of polygenic, epigenetic, metabolomic, endocrine, inflammatory and routine clinical lab markers, computerized neurocognitive testing, and symptom self-reports. The analysis was computed on active-duty Army personnel (*N* = 473) of the 101st Airborne at Fort Campbell, Kentucky. Machine-learning models predicted provisional PTSD diagnosis 90–180 days post deployment (random forest: AUC = 0.78, 95% CI = 0.67–0.89, sensitivity = 0.78, specificity = 0.71; SVM: AUC = 0.88, 95% CI = 0.78–0.98, sensitivity = 0.89, specificity = 0.79) and longitudinal PTSD symptom trajectories identified with latent growth mixture modeling (random forest: AUC = 0.85, 95% CI = 0.75–0.96, sensitivity = 0.88, specificity = 0.69; SVM: AUC = 0.87, 95% CI = 0.79–0.96, sensitivity = 0.80, specificity = 0.85). Among the highest-ranked predictive features were pre-deployment sleep quality, anxiety, depression, sustained attention, and cognitive flexibility. Blood-based biomarkers including metabolites, epigenomic, immune, inflammatory, and liver function markers complemented the most important predictors. The clinical prediction of post-deployment symptom trajectories and provisional PTSD diagnosis based on pre-deployment data achieved high discriminatory power. The predictive models may be used to determine deployment readiness and to determine novel pre-deployment interventions to mitigate the risk for deployment-related PTSD.

## Introduction

Soldiers are at risk for developing post-traumatic stress disorder (PTSD) and the lifetime prevalence of probable PTSD of 8% [1] is elevated compared with the general population (6.1%) [2]. Deployment-related PTSD risk differs from single event trauma in civilians in that active-duty military personnel are repeatedly exposed to situations in which their own life is threatened and, at times, they are required to kill enemy combatants. A better understanding of the population-specific risk factors is of great importance to mitigate modifiable risk of deployment-related PTSD that can result in burdens to the individual and society. These burdens include the suffering associated with symptoms of PTSD and its common comorbidities including depression, alcohol and drug abuse, chronic pain, and sleep disturbance. Frequent comorbidities of PTSD include metabolic syndrome, cardiometabolic, traumatic brain injury, and neurologic disease. All of these can cause disruption in relationships and work-related functions [3, 4]. In consequence, mental health needs of veterans contribute to high care usage at medical centers of the Veterans Health Administration [5, 6].

Mitigating risk for deployment-related PTSD is a complex task. A first step toward targeted PTSD prevention is to examine pre-deployment risk factors. Despite earlier skepticism [7], recent research suggests that pre-deployment risk factors can be identified and potentially mitigated [8]. Previous studies found that alterations in inflammation and metabolomics [9], as well as epigenetically altered networks [10], and a polygenic risk score (PRS) [11], are associated with the development of deployment-related PTSD. In addition, neurocognitive dysfunction [12, 13] and deployment-related data and self-reported symptoms [14], such as pre-deployment nightmares or mental health status [15] have been identified as predictive markers for the development of PTSD in soldiers who were either first time deployed or had been deployed before [16]. Based on these promising findings, we developed the rationale to examine the predictive value of all these multiple factors together in a single multivariable prognostic model of PTSD symptoms. Data-driven approaches for classification are a particularly valuable approach to combine highly multivariate data [17]. Random forest (RF) ensembles of decision trees is a data-driven machine learning (ML) approach that uses an algorithm to recursively search for an optimal model given the data. Compared with traditional statistics, where model selection is based on theoretical assumptions, ML is more flexible as numerous models are fit to describe the data and the model parameters are empirically determined. The application of methodological safeguards such as bootstrapping or cross-validation prevents the selection of a data-bound model that does not generalize to other samples (“overfitting”) [18]. In addition, a portion of the sample may be hold out to evaluate the model on separate data not used to select the model. This further corroborates the confidence in the accuracy of the results of the predictive model.

The current state of research using ML in PTSD resilience research is summarized in two recent review articles [19, 20]. In military context, a few major studies identifying risk factors using ML for predicting suicide [21], psychiatric disorders [22], and PTSD in military personnel [14, 23, 24] have been conducted. Advanced computational methodology has demonstrated that nonlinear and highly interacting combinations of heterogeneous risk factors are most predictive [23], despite the fact that such complex probabilistic information is difficult to grasp. In addition, modeling PTSD symptom development as distinct trajectories allows accounting for variability in the temporal evolution of symptoms [25]. Recognition that prediction of pre-deployment risk may be more complicated than a simplistic linear relationship of a limited number of variables with a cross-sectional risk estimate is further corroborated by recent findings of differential risk profiles associated with epigenetically altered networks [7, 10, 26, 27].

This prospective longitudinal study aims to determine whether a comprehensive set of diverse pre-selected biological, clinical, and neurocognitive variables ascertained prior to deployment is informative for predicting PTSD symptom development over the course of 90–180 days after returning from a 10-month tour of duty. In addition, we investigate whether these variables predict provisional PTSD diagnosis within 90–180 days after return. Our large multidimensional dataset consists of multi-omic blood markers including genome-wide association study (GWAS) information for a PRS, epigenomic, metabolomic, endocrine, inflammatory, and routine clinical blood tests, and computerized neurocognitive testing and symptom self-reporting measures obtained from a prospective, naturalistic, and longitudinal study cohort. This approach has the potential to discover novel pre-deployment risk factors for PTSD, to discriminate between different symptom trajectories, and may eventually contribute to the subtyping of prognostic biomarkers.

The objective is to build an accurate classification algorithm for predicting membership in PTSD symptom trajectories across three phases of the deployment cycle, and for predicting those who screen positive for a provisional PTSD diagnosis. RF was chosen for the data-driven multivariable predictive modeling [28]. For comparison, the results are benchmarked with support vector machine classifiers (SVM) [29] that have been successful for binary class prediction even on small clinical samples, such as cancer classification with *N* = 38 and 50 predictors [30]. RF is widely used to analyze large datasets such as GWAS and metabolomic data and can handle correlated predictors and nonlinear interactions and require no parametric assumptions about the underlying probability distributions. Being based on the aggregation of numerous simple decision trees [31] constructed from bootstrapped resamples [32] of the data, RF is a statistically relatively well understood ML method [33]. In addition, RF yields reliable rankings of the risk factors in order of importance for prediction [34].

We hypothesized that it is feasible to identify informative pre-deployment predictors in our dataset that will discriminate active-duty military personnel who are likely to be on an increasing PTSD symptom trajectory at post deployment from those who are not. To support clinical decision-making, we also aimed to discriminate between those who will develop PTSD symptoms above a cutoff score for provisional diagnosis versus those who will not develop clinically relevant PTSD symptom levels at 90–180 days after deployment.

## Materials and methods

### Participants

This naturalistic prospective cohort study comprised *N* = 473 active-duty Army personnel of the 101st Airborne at Fort Campbell, Kentucky, assessed before and after being deployed to Afghanistan in February 2014 (index deployment). GWAS data of 1600 participants were available to calculate a PRS to use for this study. Participants were either first time deployed (*n* = 272) or had previously been deployed before once (*n* = 102), twice (*n* = 52), or more than two times (*n* = 47). The first phase of recruitment occurred during a 2-week period immediately prior to deployment in February 2014. The second phase occurred 3 days after returning from a 10-month tour of duty. The third phase occurred 90–180 days post deployment. The inclusion and exclusion criteria are presented in the supplementary methods. The Fort Campbell Cohort (FCC) study was designed to identify PTSD risk factors and was conducted in accord with ethical principles for the conduct of human research as specified in the Declaration of Helsinki [35]. The Institutional Review Board of NYU Grossman School of Medicine, approved the study, as well as the Human Research Protection Office of the United States Army at Fort Detrick, Maryland and Army Command of the 101st Airborne at Fort Campbell, Kentucky. All participants signed the informed consent. Reporting guidelines for cohort studies [36] and recommendations for ML [37] were followed as applicable.

### Procedure

This prospective longitudinal study comprised three phases, from which all participants who had available scores of the PTSD Checklist for DSM-5 (PCL-5) [38] at Phase 1 and Phase 3 were included (see flow chart in Supplementary Fig. 1).

### Data collection

In contrast to many other prospective longitudinal studies of stress-exposed cohorts, this study assessed participants prior to stressor exposure during the index deployment and included gender, age, race and education, and military service information along with comprehensive whole blood, plasma, serum, and buffy-coat biomarkers as well as clinical self-report and neurocognitive functioning measures. We included 105 candidate predictors based on prior theory. A complete overview with basic descriptive statistics is presented in Supplementary Table 1.

### Clinical assessment: psychological symptoms and functioning

We collected self-reports of symptoms and functioning, using the PCL-5 [38], the Patient Health Questionnaire (PHQ-8) to measure symptoms of depression [39], Generalized Anxiety Disorder (GAD-7) [40] and the Alcohol Use Identification Test to measure alcohol abuse [41]. The Ohio Traumatic Brain Injury Assessment was used to ascertain traumatic brain injury [42], the Pittsburgh Sleep Quality Index for capturing current sleep quality [43], and the Concussion Symptoms Inventory to assess lifetime concussion (symptoms for the month in which concussive symptoms were the worst) and current post-concussive symptoms during the past month [44]. In addition, the Deployment Risk and Resilience Inventory-2 (DRRI-2) was assessed for determining warzone exposure [45].

### Cognitive assessment: attention, emotion regulation, and executive function

We used a computerized neurocognitive assessment tool (WebNeuro) to assess cognitive and emotional functioning, including information processing, working memory, emotion regulation, and psychomotor functioning. We included measures of sustained attention, inhibitory control, cognitive flexibility, and processing speed in our predictive models [46].

### Blood draw: multi-omics including routine clinical labs

Multi-omics data were included based on previous findings from The PTSD Systems Biology Consortium [24, 47]. We assessed LabCorp Clinical Laboratory Improvement Amendments-certified lab tests for complete blood count (CBC). In addition, we assessed lipid panel, inflammatory markers and liver functioning tests, metabolomics and methylation marks as well as a PRS for PTSD [11].

### Statistical analysis

#### Latent growth mixture modeling (LGMM)

All participants in the sample (*N* = 473) who completed the PCL-5 for both Phase 1 and 3 were included in latent growth mixture modeling (LGMM) using also available Phase 2 scores. LGMM was fit in Mplus version 7 [25]. To determine how many distinct latent classes best described the trajectories of PTSD symptom severity in FCC samples, a series of LGMM models were constructed. The best-fitting model was identified using recommendation from the literature [48].

#### Predictive models

The dependent variable for classification was the assignment to exactly one LGMM class. A second RF and SVM model was developed to predict two groups, those who met and those who did not meet the PCL-5 cutoff score for a provisional PTSD diagnosis at Phase 3, which occurred 90–180 days post deployment. A PCL-5 total score of ≥31 was defined as the cutoff for screening positive for a provisional diagnosis of PTSD in active-duty military personnel [49, 50]. The specifier “provisional” in DSM-5 was used according to the definition in the DSM-5 [51] and from the National Center for PTSD [38]. PCL-5 shows “good diagnostic utility for predicting a CAPS-5 PTSD diagnosis” and “good structural validity, and sensitivity to clinical change comparable to that of a structured interview” [52]. It shows good reliability, convergent, concurrent, discriminant, and structural validity [52].

We evaluated the training performance in terms of confusion matrix, sensitivity, and specificity, and selected the “best” model in terms of area under the receiver operating characteristic curve (AUC). All steps of data inspection and preprocessing, including imputation and analysis, were performed using R version 3.5.1 in Rstudio 1.1.456. Categorical variables were converted to binary numerical values (“dummy coding”), and missing values were imputed using bagged decision trees [18]. Twelve variables with values of near-zero variance and six variables with more than 45% missing data were removed in order to increase the accuracy of the bagged CART tree imputation. In total, 15% of the data were missing. Training and test sets were imputed separately to avoid information leakage [53]. The dependent variable was removed from the dataset prior to imputation for the same reason. The total sample was randomly split into a 75% partition as a training set to build the model and a 25% test set to evaluate the predictive power of the final model in unseen cases (Table 1). The size of the test set was adequately powered to detect an above-chance AUC > 78 with alpha = 0.05 and 90% power [54]. To balance the dependent variable across data partitions, stratified random sampling was applied [53]. The bootstrap method was used to guard against overfitting while fine-tuning the model [55] and the process was 25 times repeated to obtain robust training error estimates. For RF models (ranger R package), we used 1000 trees per forest to obtain robust permutation-based variable rank scores [34]. The parameter “minimum node size” was fixed at 1 and the number of randomly selected predictors per split and the type of splitting rule were fine-tuned by examining 100 random combinations. For SVM models, the model parameters sigma and cost were fine-tuned with a random search of 100 different combinations, with all other parameters set to default values (kernlab R package). The code is freely available upon request but for research purposes only. Supplementary Fig. 2 provides a basic schematic representation of the predictive analytics approach.

**Table 1:** Classification task and observed positive and negative events for each outcome. The term “positive events” refers to the outcome-of-interest, i.e., to those participants in the sample who are on an “increasing” PTSD symptom trajectory or who meet the cutoff for provisional PTSD diagnosis (PCL-5 score ≥31). “Negative events” are those participants who are on a “resilient” trajectory or who do not meet the cutoff. Depicted are the sample size for each outcome for the training set, the test set and the total sample.

Classification task	Training set (75%)	Test set (25%)	Total (*N* = 473)
PTSD symptom trajectories			
“Increasing”	*n* = 33	*n* = 10	*n* = 43
“Resilient”	*n* = 323	*n* = 107	*n* = 430
PCL-5 cutoff score			
Provisional PTSD	*n* = 27	*n* = 9	*n* = 36
No PTSD	*n* = 328	*n* = 109	*n* = 437

#### Predictor importance ranking

Variables included in the final models were ranked with respect to their ability to predict both PTSD symptom trajectory membership across the three phases and PTSD case status at Phase 3 using a permutation procedure along with *p* values (see Supplementary material for details) [28, 34].

## Results

Descriptive statistics on the sample characteristics at Phase 1 are presented in Table 2 (see Supplementary Table 2 for sample characteristics at Phase 3).

**Table 2:** Sample characteristics at Phase 1 of those participants included into the analysis.

	Phase 1			
	“Increasing” trajectory (*N* = 43)	“Resilient” trajectory (*N* = 430)	Provisional PTSD (*N* = 36)	No PTSD (*N* = 437)
Age	27.16 (5.99)	25.66 (5.92)	26.67 (6.15)	25.73 (5.92)
Gender (% females)	11.6% (*N* = 5)	5.3% (*N* = 23)	13.9% (*N* = 5)	5.3% (*N* = 23)
PCL-5 score	14.12 (16.66)	2.63 (5.37)	13.83 (17.17)	2.84 (5.83)
PHQ-8 score	4.5 (5.13)	1.31 (2.49)	4.54 (5.33)	1.36 (2.55)
GAD-7 score	5.55 (5.42)	1.65 (2.64)	5.4 (5.57)	1.72 (2.75)
AUDIT score	2.43 (3.28)	2.23 (2.50)	2.58 (3.57)	2.22 (2.48)
PSQI score	8.46 (3.79)	4.88 (2.9)	8 (3.63)	4.97 (3.01)
DRRI-2 score	51.7 (22.5)	35.94 (16.5)	50.86 (23.73)	36.52 (16.86)
TBI status: improbable	63.4% (*N* = 26)	84.6% (*N* = 356)	61.8% (*N* = 21)	84.3% (*N* = 361)
TBI status: possible	12.1% (*N* = 5)	5.7% (*N* = 24)	14.7% (*N* = 5)	5.6% (*N* = 24)
TBI status: mild	14.6% (*N* = 6)	8.1% (*N* = 34)	11.8% (*N* = 4)	8.4% (*N* = 36)
TBI status: moderate	9.8% (*N* = 4)	1% (*N* = 4)	11.8% (*N* = 4)	0.9% (*N* = 4)
TBI status: severe	0% (*N* = 0)	0.7% (*N* = 3)	0% (*N* = 0)	0.7% (*N* = 3)
CSI current	15.44 (12.22)	5.73 (8.92)	16.15 (12.82)	6.01 (9.02)
CSI lifetime	27.94 (18.72)	12.35 (15.2)	25.69 (15.21)	13.45 (16.7)
Number of previous deployments	1.09 (1.54)	0.76 (1.11)	0.81 (1.31)	0.79 (1.15)

*PCL-5* PTSD Checklist for DSM-5, *PHQ-8* Patient Health Questionnaire, *GAD-7* Generalized Anxiety Disorder; *AUDIT* Alcohol Use Identification Test, *PSQI* Pittsburgh Sleep Quality Index, *DRRI-2* Deployment Risk and Resilience Inventory-2, *TBI* traumatic brain injury, *CSI* Concussion Symptoms Inventory (current (past month) and lifetime (month in which symptoms were their “worst”)).

**Fig. 1:**
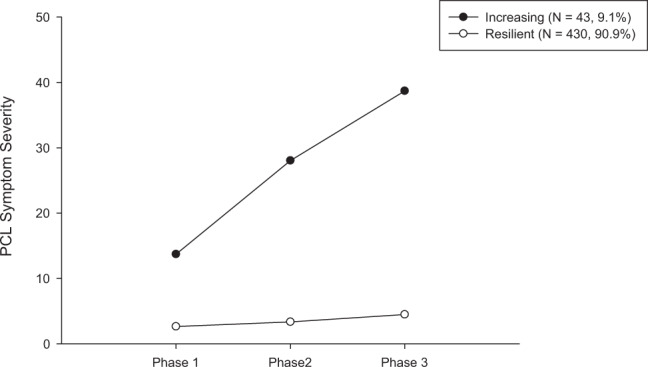
Unconditional model for the latent trajectories of the longitudinal PTSD symptom development based on PCL-5 scores through 90–180 days (Phase 1, 2, and 3). The term “unconditional” means that there are no covariates included in this LGMM model but only the PCL-5 scores (outcome-of-interest) [25]. A two-class solution with fixed slope and linear weights was identified as the best-fitting model with an entropy of 0.98 (see Supplementary Tables 3, 4). We chose linear rather than quadratic solutions for trajectories because a minimum of four time points is recommended to fit quadratic solutions. Those two trajectories can be qualitatively described as “increasing” trajectory (*N* = 43, 9.1%) and as “resilient” trajectory (*N* = 430, 90.9%).

**Fig. 2:**
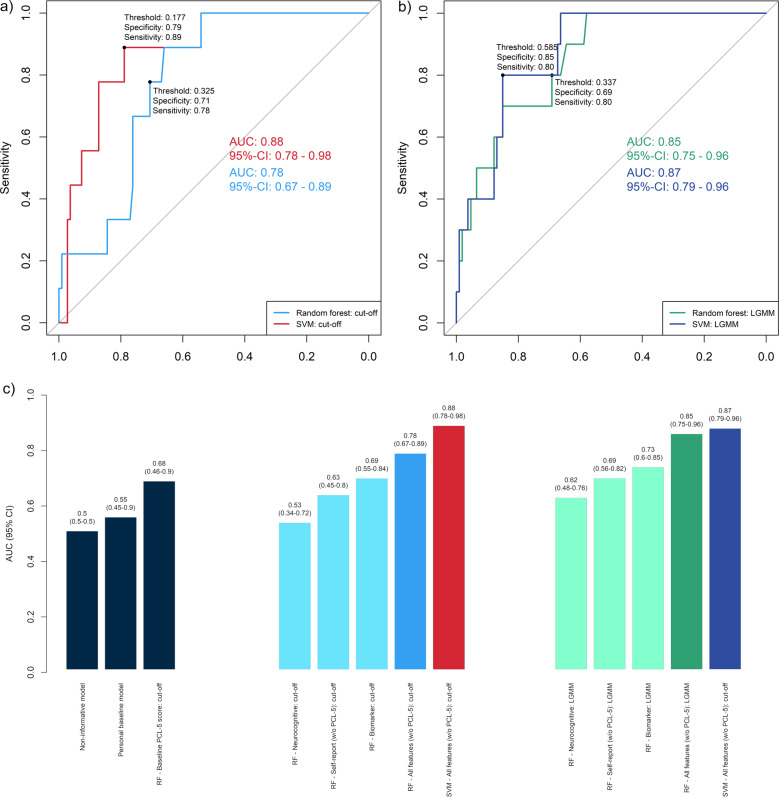
Discriminatory power of RF and SVM using different data types as predictor variables. Receiver operating characteristic curve (ROC) for the prediction of the provisional PTSD diagnosis post deployment (**a**) and of PTSD symptom trajectories (**b**) using genetic, metabolomic, methylation, inflammation, neuropsychological, and clinical data collected prior to deployment. Depicted is the optimal ROC thresholds for sensitivity and specificity as determined by min((1 − sensitivities)^2^ + (1 − specificities)^2^), which yields the threshold closest to the top-left corner of the ROC curve [73]. DeLong’s test for two correlated ROC curves [74] shows no significant difference between the RF and SVM models for predicting LGMM trajectories (*Z* = 0.403, *p* = 0.3435), but significant differences for provisional PTSD diagnosis (*Z* = 1.7587, *p* = 0.03932). The bar plot (**c**) displays the comparison of the predictive models with different benchmark models. All four models (SVM and RF models predicting provisional PTSD diagnosis and SVM and RF models predicting PTSD symptom trajectories) have significantly higher discriminatory power than a non-informative model that predicts all participants as “PTSD negative,” i.e., is low or subthreshold PTSD symptoms (see Supplementary Table 7). All four models are significantly better than a benchmark model using a subject-specific baseline score as predicted outcome [56], i.e., using the individual pre-deployment PTSD status as indicated by the PCL-5 (see Supplementary Table 14).

The predictive model based on pre-deployment neurocognitive, psychometric self-report, and biomarker information from a total of 473 participants showed high discriminatory power to distinguish PTSD symptom severity trajectories for 90–180 days post deployment (Fig. 1; Supplementary Tables 3 and 4 display the results of the LGMM). Using the RF algorithm, the averaged out-of-bag result on the training dataset was AUC = 0.79 (SD = 0.07). On the internal test set, the performance was confirmed, with the 95% CI of the AUC of 0.75–0.96 (AUC = 0.85, sensitivity = 0.80; specificity = 0.69; see Fig. 2). The discriminatory power further increased using SVM models (Fig. 2).

The RF algorithm was also able to predict provisional PTSD diagnosis at Phase 3 based on pre-deployment data (AUC = 0.78 for the internal test set, with 95% CI of 0.67–0.89; sensitivity = 0.78; specificity = 0.71; see Fig. 2). The averaged out-of-bag result for provisional PTSD diagnosis on the training dataset was AUC = 0.78 (SD = 0.08).

Similar to the lifetime prevalence of probable PTSD of 8% in US Veterans [1], 7.6% of the participants screened positive for provisional PTSD diagnosis (PCL-5 cutoff ≥ 31) [49, 50]. A notable 92.4% reported no or only few PTSD symptoms at Phase 3. Due to the resulting class imbalance in the outcome, we present additional evaluation metrics in the Supplementary Tables 5–11 and Supplementary Figs. 3 and 4 including precision–recall curves. One-sided DeLong’s test showed that both RF models were significantly better in discriminating between the outcomes-of-interest compared with a non-informative model, which assigns all participants to the majority class (LGMM trajectories as the outcome: *Z* = 6.6476, *p* = 1.489e−11; provisional PTSD diagnosis as outcome: *Z* = 4.9214, *p* = 4.297e−07). Figure 2c shows the comparison between the AUC, including 95% CI, of the RF and SVM models with different “population-based” and “personal” benchmark models [56].

In addition, there were significant differences in warzone exposure during index deployment (section D “Combat Experiences” of the DRRI-2). The participants on the “increasing” trajectory experienced significantly more traumatic events during combat (*t*(248) = 2.85, *p* = 0.005; “increasing” trajectory; mean = 28.89 ± 10.84; “resilient” trajectory; mean = 23.90 ± 7.01). The same was true for those participants with the provisional PTSD diagnosis (*t*(248) = −3.23, *p* = 0.001; provisional PTSD; mean = 30.20 ± 11.76; no PTSD; mean = 23.90 ± 6.97).

Supplementary Table 12 shows the results of the Pearson’s *χ*^2^-test with Yates’ continuity correction to show that missing values were not significantly more frequent in more severe PTSD cases. Supplementary Figure 5 represents the results of a different train-test split to show that the results are robust and Supplementary Fig. 6 displays the model to predict PTSD development using only the PCL-5 subitems and total score at Phase 1.

### Ranking the risk variables for predictive value

**Fig. 3:**
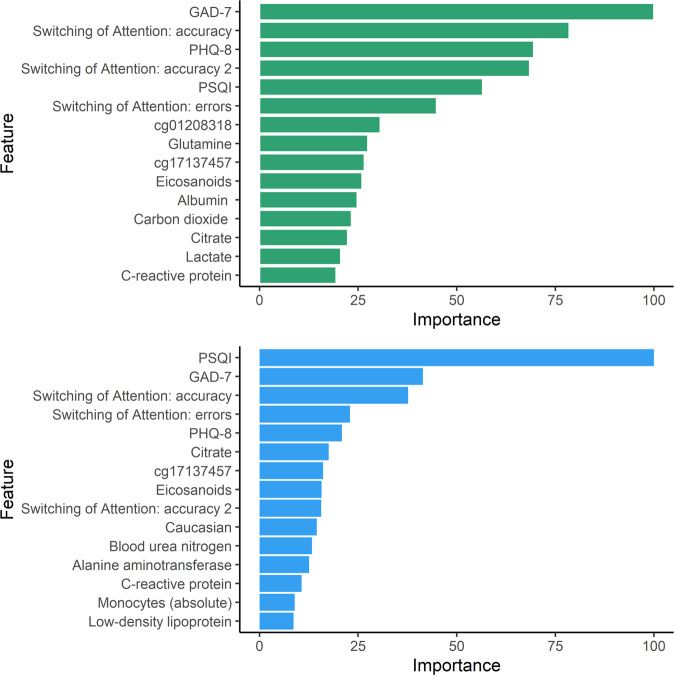
Display of the top 15 predictor variables for predicting LGMM trajectories (green bars) and for predicting provisional PTSD diagnosis (blue bars). In permutation-based ranking [34], the importance of a feature is measured by calculating the increase in the model’s prediction error after reshuffling the distribution of the feature values. The *y*-axis presents the importance ranking, with the top features being the most important ones. The *x*-axis denotes the classification error scaled to range 0 to 100. It is not recommended to interpret the absolute importance value, but only the rank order between features [75]. All features shown in Fig. 3 contributed significantly (*p* < 0.01) to the respective predictive model [34]. Statistical significance is indicated by the bias-correcting PIMP algorithm, which tests the importance of each predictor under the distribution of *“*null importance” values derived for every variable from 100 permutations of the response variable [34].

Predictive performance was best when multiple heterogeneous sources of information were integrated into a comprehensive model (Fig. 2c). Figure 3 displays the top 15 predictor variables ranked using permutation-based variable importance approach [34].

The pairwise correlation of the top 15 features is shown in Supplementary Fig. 7. Significant univariate mean group differences for both outcomes are presented in Supplementary Table 13. Supplementary Figures 8 and 9 display further variable importance metrics. Supplementary Figure 10 visualizes the variable importance for classifying those who fulfill the criteria for depression according to PHQ-8 and for depression and PTSD. Supplementary Figure 11 displays the most important features for the SVM model for predicting provisional PTSD diagnosis. Supplementary Figure 12 presents the results of a regularized RF using all subitems of the self-report instruments. Supplementary Figure 13 displays the heatmap for the correlation matrix of the subitems of the psychometric instruments.

## Discussion

Among active-duty military personnel deployed to Afghanistan, we found that pre-deployment risk factors predicted PTSD symptom trajectories and provisional PTSD diagnosis 90–180 days after returning from the deployment. Our results provide evidence that pre-deployment PTSD risk can be predicted based on the combination of biomarkers, self-reports, and neurocognitive functioning. Using this information, the overall best prediction model (SVM) discriminates post-deployment LGMM trajectories and provisional PTSD diagnosis with high sensitivity and specificity (Fig. 2). Both RF and SVM models performed significantly better than a non-informative benchmark model that assigns all participants the same constant prediction of the majority class, i.e., “no PTSD,” (Supplementary Table 7) and a benchmark model using the subjects individual pre-deployment PTSD status based on the pre-deployment PCL-5 score as a person-specific baseline prediction for each subject (Supplementary Tables 14–17). Overall, the SVM model performed significantly better than all benchmark models (Fig. 2c and Supplementary Tables 14–17). The test-set size was powered to reliably detect an AUC of the size of the AUC of the RF and SVM model. As a limitation, it should be noted that for models with an AUC < 0.75 (Fig. 2c), the power of the test set is limited. While the 95% CI indicates substantial overlap, the width of the CI depends on the sample size and a larger test set would be needed to prevent Type-II errors (i.e., falsely assuming there is no difference in performance) when comparing these models. While future research may determine whether differences in pre-deployment PTSD symptom status, biomarkers, self-reports, and neurocognitive functioning are sufficiently predictive in isolation, we report evidence that the combination of this data is best predictive overall (Fig. 2).

The main result that the combination of pre-deployment factors provides predictive information about post-deployment PTSD risk is consistent with the diversity of prognostic factors previously reported in the PTSD literature [4, 9, 10, 57]. However, previous studies are often cross-sectional, making it difficult to differentiate risk factors from consequences of developing PTSD [58] or do not include comprehensive biomarker information [14].

Beyond the FCC study, only a few large prospective longitudinal studies analyzed risk factors for PTSD in military personnel, such as the Dutch Prospective Research in Stress-related Military Operations (PRISMO) study [59, 60], the UK Air Force cohort of King’s Centre for Military Health Research [14], and the US Marine Resiliency Studies (MRS, MRS-II) [61].

Recently, a multi-omics panel of 28 biomarkers was derived from more than 300 biomarkers as candidate diagnostic biomarkers for PTSD [24]. Among the top 10 predictors of this study [24] are four biomarkers (cg01208318, cg17137457, lactate, citrate) that are also in the top 15 of our study (Fig. 3). In the current study, we show the predictive relevance of the biomarkers when combined with self-reported clinical symptoms and neurocognitive functioning (Fig. 2).

### Biomarkers

Peripheral inflammatory and immune markers in the blood, such as monocytes, basophil, and C-reactive protein (CRP), were found to be important predictors. This fits with previous cross-sectional findings of altered mitochondrial function [47] and the finding in the MRS cohort that higher plasma levels of CRP prior to deployment predicted the development of deployment-related PTSD [62]. Similar to the monocytes and basophil in our sample, the PRISMO study demonstrated that leukocyte sensitivity to glucocorticoids (high dexamethasone-sensitivity of T-cell proliferation) prior to deployment was associated with post-deployment PTSD, but only in those without comorbid depression symptoms [60].

There is mounting evidence about the crosstalk of inflammatory responses of the immune system and mitochondrial function and metabolic markers of mitochondrial dysfunction may be associated with PTSD [9]. We found that mitochondrial metabolites including lactate, citrate, eicosanoids, and glutamine were highly ranked predictive features. In line with previous studies, we also found that citrate was decreased in PTSD subjects [47].

Moreover, epigenomic mechanisms may explain gene by environment interactions in PTSD that contribute to increased risk or resilience. We found that mitochondria-related DNA methylation (cg17137457) of the CPT1B gene contributes to the prediction of provisional PTSD. CPT1B is overexpressed in the amygdala in a rodent PTSD-model and also in the blood of humans with PTSD where it is acting on fatty acid metabolism [63]. In addition, we found a predictive relevance of a lipid panel including LDL cholesterol, which may suggest an association of PTSD risk with metabolic dysregulation as previously reported [9].

Contrary to our expectation, our PRS was not among the most relevant predictors. Previous GWAS studies found mixed results [64, 65] but recently new loci have been suggested [66] and further research is necessary.

Finally, previous studies [67, 68] suggested that the pre-deployment cortisol awakening response [68] and hair cortisol [67] predict post-deployment PTSD symptoms. In our sample, we did not identify pre-deployment plasma cortisol levels among the most important predictors. Further research is needed to examine if the prediction can be further improved by assessing hair cortisol or the cortisol awakening response instead of plasma cortisol levels.

### Neurocognitive function

Computerized neurocognitive measures of cognitive flexibility and sustained attention were predictors for PTSD, which fits prior findings [12, 13] that indicated cognitive flexibility [69] and sustained attention [70] as relevant pre-deployment predictors of deployment-related PTSD.

### Psychometric assessment

Similar to the Millennium Cohort Study [15], we found that self-reported anxiety (GAD-7) and depressive symptoms (PHQ-8) are highly ranked predictors of deployment-related PTSD. A recent study using ML emphasizes the importance of self-reported symptoms for classifying PTSD caseness [14]. In line with the PRISMO study [59], we also found that self-reported sleep quality prior to deployment ranked high among the predictors of post deployment. This is well-aligned with results of a longitudinal study (*N* = 561) of Danish soldiers deployed in Afghanistan in 2006 [23] in which psychometric together with sociodemographic information was strongly predictive in classifying PTSD symptom trajectories using SVM (AUC = 0.84; 95% CI = 0.81–0.87) [23].

### Strength and limitations

This naturalistic, prospective longitudinal cohort study has high external and internal validity since the study was designed around a cohort that experienced combat zone-deployment as a shared potential stressor [71]. Our study design resembles other naturalistic cohort studies that investigate shared stress exposures and heterogeneous trajectories of psychopathology [23]. This study provides a comprehensive set of biological, clinical, and cognitive predictors to investigate multivariate risk prior to deployment. A limitation is the inclusion of only those individuals for whom PCL-5 scores were available at Phases 1 and 3. In addition, provisional PTSD diagnosis needs to be verified using the SCID or CAPS. Furthermore, external validation in independent datasets is necessary to assess the generalizability of the model. The possibility of potential unknown confounders should be acknowledged as in any naturalistic cohort study. The reported associations among the predictors and the PTSD symptom trajectories and provisional diagnoses should not be interpreted causally. The candidate predictors require experimental manipulations to test for causal determination of risk.

### Clinical implications

The assessment of active-duty Army personnel at pre-deployment and after deployment using biological and behavioral measurement enables us to identify pre-deployment risk factors for the development of deployment-related PTSD. The identified risk factors can be used to inform deployment readiness, e.g., using self-report measures along with inexpensive blood testing for CBC or CRP, and to target preventive interventions for improving the resilience of military personnel. Pre-deployment self-reported information including differences in stress symptoms, sleep problems, anxiety, and depressive symptoms are predictors that are low cost, easily ascertained, and indicate modifiable factors.

## Conclusions

The biological, clinical, and neurocognitive assessment of military personnel before deployment raises the possibility of predicting post-deployment PTSD risk to inform future research on risk factors and to inform the planning of targeted prevention of deployment-related PTSD. Our modeling approach acknowledges the complex nature of current theories of PTSD [72] spanning from various molecular (e.g., genetic, metabolomic, immunologic, and neurobiological) levels of explanation to multiple high-level systems of causal pathways, including cognitive domains and social environments. This approach is promising for future work on individualized risk prediction and prevention.

## Disclaimer

The views, opinions, and/or findings contained in this report are those of the authors and should not be construed as official Department of the Army position, policy, or decision, unless so designated by other official documentation. Citations of commercial organizations or trade names in this report do not constitute an official Department of the Army endorsement or approval of the products or services of these organizations. Opinions, interpretations, conclusions, and recommendations are those of the authors and are not necessarily endorsed by the US Army.

## Supplementary information

Supplementary Material
