# Supplementary material for: Pre-deployment risk factors for PTSD in active-duty personnel deployed to Afghanistan: a machine-learning approach for analyzing multivariate predictors
Source: Mol Psychiatry. 2020 Jun 2;26(9):5011–22. doi: 10.1038/s41380-020-0789-2 (PMC8589682; doi:10.1038/s41380-020-0789-2)
Supplement: Supplementary file 1 — Supplementary Material [file 41380_2020_789_MOESM1_ESM.docx]

# **Supplementary Material**

# Pre-deployment risk factors for PTSD in Afghanistan active duty personnel: a machine learning approach for analyzing multivariate predictors

Katharina Schultebraucks, PhD, Meng Qian, PhD, Duna Abu-Amara, Kelsey Dean, Eugene Laska, PhD, Carole Siegel, PhD, Aarti Gautam, PhD, Guia Guffanti, PhD, Rasha Hammamieh, PhD, Burook Misganaw, PhD, Synthia H. Mellon, PhD, Owen M. Wolkowitz, MD, Esther Blessing, MD, PhD, Amit Etkin, MD, PhD, Kerry J. Ressler, MD, PhD, Francis J. Doyle III, PhD, Marti Jett, PhD, Charles R. Marmar, MD

KS: Department of Psychiatry, New York University School of Medicine, New York; Vagelos School of Physicians and Surgeons, Department of Emergency Medicine, Columbia University Medical Center, New York

EL, CS, MQ, DAA, EB, CRM: Center for Alcohol Use Disorder and PTSD, Department of Psychiatry, New York University School of Medicine, New York

GG, KJR: McLean Hospital, Harvard University, Boston, Massachusetts and Department of Psychiatry and Behavioral Sciences, Emory University School of Medicine, Atlanta, Georgia

AE: Department of Psychiatry, Stanford University, Palo Alto, California

AG, RH, MJ: Integrative Systems Biology, US Army Center for Environmental Health Research, USACEHR, Fort Detrick, Frederick, MD

KD, FJD: Harvard Paulson School of Engineering & Applied Sciences, Boston, Massachusetts

SHM: Department of Obstetrics, Gynecology & Reproductive Sciences, University of California, San Francisco

OMW: Department of Psychiatry, University of California, San Francisco

Here we provide further details about the analytic rationale of the study and the results. Although this information may be relevant for replication and future research, the strict word limit did not allow the inclusion into the main text.

**Supplementary Methods**

**Participant flow**

In Phase 1, N=1,029 active duty Army personnel were assessed immediately prior to deployment to Afghanistan. In Phase 2, N=760 participants were assessed three days after return from Afghanistan, of which N=506 participated in Phases 1 and 2 and 204 were newly recruited and were included in Phase 2. In Phase 3, N=1,166 participants were assessed 90 to 180 days after deployment, including N=731 participants who were assessed in Phase 1 and Phase 2 and/or 3. In total, N=1,793 unique Army personnel were enrolled in one or more of the three phases. The prospective cohort was enriched by participants newly included in Phases 2 and 3. These additional participants were also deployed to Afghanistan during the index deployment in 2014, but chose not to participate in Phase 1. Active duty personnel, who were deployed to Afghanistan of all races and ethnic origin whose primary language is English and are able to understand the protocol and willing to provide written informed consent were eligible for participation in this study. In the current study we included all participants who had available scores of the PTSD Checklist for DSM-5 (PCL-5) ^1^ at Phase 1 and Phase 3.


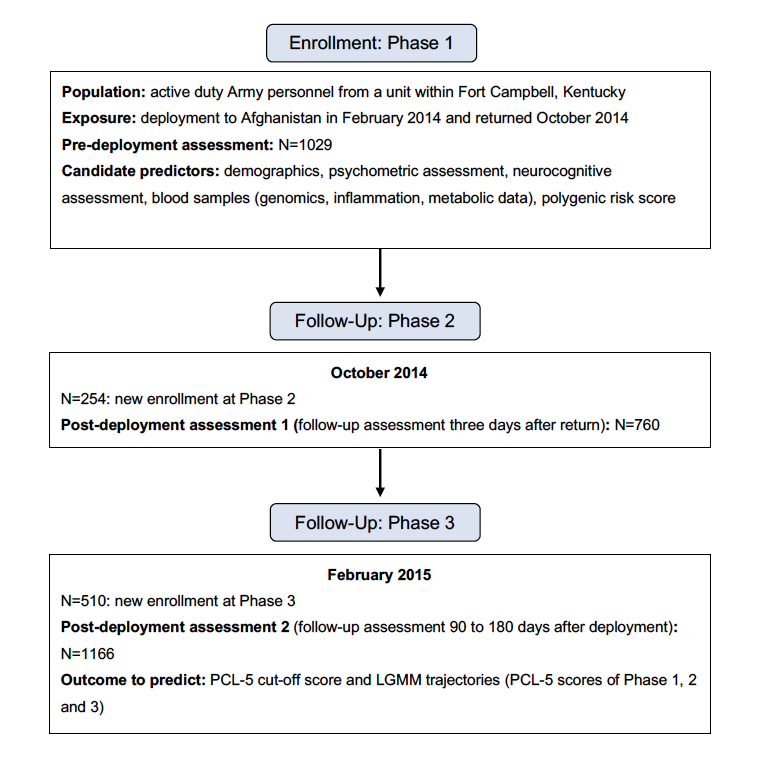


Supplementary Figure 1. Schematic overview of study procedure and study flow.

***GWAS Analysis***

Genotype data were imputed using Phase 3 1000 genomes data as a reference panel and SHAPEIT and IMPUTE statistical programs. Imputed data were further screened to ensure the quality of imputation procedure. In particular, SNPs were filtered for imputation quality > 0.8, MAF < 0.01, and genotyping rate < 0.05. The final imputed dataset includes ~ 6 M SNPs available for downstream analysis.

Genotyping was completed for over N=1,600 participants using the Illumina Infinium PsychArray BeadChips. Specifically, this chip includes 265,000 proven tag SNPs found on Illumina’s HumanCore BeadChip, 245,000 markers from the HumanExome BeadChip, and 50,000 markers associated with common psychiatric disorders.

PRSice analysis ^2^ was conducted using the standard protocol of LD clumping followed by significance thresholding procedure. Each polygenic risk score (PRS) analysis was performed using the top 5/10 ancestry-related principal components to correct for hidden population stratification. The LD clumping was performed using windows of 250 kb, by which the program clumps any SNPs within 250 kilobyte (Kb) upstream or downstream from the index SNP, using a squared correlation of r^2^ = 0.1 for clumping. PRS was calculated for different p-value thresholds ranging from 0.1 to 1 and for each PRS a regression was performed to test the association of the PRS with the phenotype. Nagelkerke R^2^ and nominal significance of the association of the PRS with the phenotype were used as criteria to define the PRS with the highest predictive value.

Supplementary Table 1. Descriptive statistics of the included features in the final model.

| **FCC cohort study (N=473) at Phase 1 (prior to index deployment)** | | | | |
| --- | --- | --- | --- | --- |
| **Feature group** | | **Feature name** | **Feature value** | **Units** |
| **Demographics** | | Gender (% female) | 5.9% |  |
|  |  | Age (mean +/-SD) | 25.80 (5.94) | Years |
|  |  | Race (% |  | Categorical |
|  |  | Asian | 1.5% |  |
|  |  | Black or African American | 14.2% |  |
|  |  | White or Caucasian | 64.5% |  |
|  |  | Native American | 1.5% |  |
|  |  | Hispanic or Latino | 14% |  |
|  |  | Other | 4.4% |  |
|  |  | Education |  | Categorical |
|  |  | Up to 12^th^ grade | 3.4% |  |
|  |  | H.S. Diploma or GED (12 yrs.) | 62.6% |  |
|  |  | 2 yrs. college or A.A. degree | 20.5% |  |
|  |  | 4 yrs. college or Bachelor’s degree | 10.4% |  |
|  |  | Master’s degree | 2.3% |  |
|  |  | Doctoral degree | 0.8% |  |
|  |  | Military service information |  | Categorical |
|  |  | First time deployment | 57.5% |  |
|  |  | Second re-deployment | 21.6% |  |
|  |  | Third re-deployment | 11% |  |
|  |  | Fourth re-deployment | 2.7% |  |
|  |  | Fifth re-deployment | 1.1% |  |
|  |  | Sixth re-deployment | 0.2% |  |
| **Clinical Assessment:** Psychological Symptoms and Functioning |  | Patient Health Questionnaire  (PHQ-8), (mean +/-SD) | 1.60 (2.96) | [0-24] |
|  |  | Generalized Anxiety Disorder  (GAD-7), (mean +/-SD) | 2 (3.20) | [0- 21] |
|  |  | Alcohol Use Identification Test (AUDIT), (mean +/-SD) | 2.24 (2.58) |  |
|  |  | Ohio Traumatic Brain Injury Assessment, (mean +/-SD) | 0.34 (0.84) |  |
|  |  | Concussion Symptoms Inventory (CSI), (% yes) | 17.8% |  |
|  |  | CSI current (mean +/-SD) | 7.62 (10.32) |  |
|  |  | CSI lifetime (mean +/-SD) | 15.39 (16.99) |  |
|  |  | Pittsburgh Sleep Quality Index (PSQI), (mean +/-SD) | 5.19 (3.15) | [0- 21] |
|  |  | Deployment Risk and Resilience Inventry-2 (DRRI-2), (mean +/-SD) | 37.54 (17.75) |  |
| **Cognitive Assessment:** Attention, Emotion Regulation, Executive Function | WebNeuro | Wmfpk: Sustained Attention - Errors of Commission(mean +/-SD) | 7.05 (13.90) |  |
|  |  | Wmfnk: Sustained Attention - Errors of Omission (mean +/-SD) | 1.10 (1.65) |  |
|  |  | Wmrtk: Sustained Attention - Reaction Time (mean +/-SD) | 524.59 (128.49) |  |
|  |  | Wmsdk: Sustained Attention - Variability of Reaction Time  (mean +/-SD) | 178.09 (123.32) |  |
|  |  | Wmerrk: Sustained Attention - Total Errors (mean +/-SD) | 8.14 (14.16) |  |
|  |  | Swoaact2: Switching of Attention – accuracy (mean +/-SD) | 96.93 (6.08) |  |
|  |  | Swoadut2: Switching of Attention - Completion Time  (mean +/-SD) | 47028.71 (14807.82) |  |
|  |  | Esoadur2: Switching of Attention - Completion Time (digits + letters), (mean +/-SD) | 46753.81 (14812.37) |  |
|  |  | Esoaerr2: Switching of Attention – errors (digits + letters), (mean +/-SD) | 1.05 (2.41) |  |
|  |  | Swoaac2: Switching of Attention – accuracy (mean +/-SD) | 94.03 (11.62) |  |
| **Blood Draw**: Multi-omics including routine clinical labs | LabCorp Clinical Laboratory Improvement Amendments | Hemoglobin (mean +/-SD) | 14.78 (1.07) | g/dL |
|  |  | glycated hemoglobin (hemoglobin A1C), (mean +/-SD) | 5.37 (0.28) | % |
|  |  | Hematocrit (mean +/-SD) | 43.62 (2.77) | % |
|  |  | Immature Granulocytes absolute (mean +/-SD) | 0.001 (0.01) | x10E3/uL |
|  |  | Immature Granulocytes  (mean +/-SD) | 0.02 (0.17) | % |
|  |  | Mean cell hemoglobin (mean +/-SD) | 29.80 (1.67) | pg |
|  |  | Mean corpuscular hemoglobin concentration (mean +/-SD) | 33.88 (0.96) | g/dL |
|  |  | Mean corpuscular volume  (mean +/-SD) | 87.99 (4.24) | fL |
|  |  | Platelets (mean +/-SD) | 256.74 (51.63) | x10E3/uL |
|  |  | Red blood cell count (mean +/-SD) | 4.97 (0.36) | X10E6/uL |
|  |  | Red blood cell distribution width (mean +/-SD) | 13.60 (0.69) | % |
|  | Metabolic | Albumin (mean +/-SD) | 4.77 (0.26) | g/dL |
|  |  | Albumin to Globulin (A/G) ratio (mean +/-SD) | 1.78 (0.23) | 1 |
|  |  | Globulin total (mean +/-SD) | 2.71 (0.31) | g/dL |
|  |  | Total bilirubin (mean +/-SD) | 0.60 (0.32) | mg/dL |
|  |  | Blood urea nitrogen (BUN),  (mean +/-SD) | 14.61 (3.63) | mg/dL |
|  |  | BUN creatinine ratio (mean +/-SD) | 15.30 (3.67) | 1 |
|  |  | Creatinine (mean +/-SD) | 0.96 (0.14) | mg/dL |
|  |  | Calcium (mean +/-SD) | 9.79 (0.34) | mg/dL |
|  |  | Carbon dioxide (CO_2_), (mean +/-SD) | 23.47 (1.67) | mmol/L |
|  |  | Chloride (mean +/-SD) | 100.68 (1.89) | mmol/L |
|  |  | Glucose (mean +/-SD) | 84.07 (10.63) | mg/dL |
|  |  | Protein (mean +/-SD) | 7.48 (0.40) | g/dL |
|  |  | Sodium (mean +/-SD) | 139.99 (1.80) | mmol/L |
|  |  | Potassium (mean +/-SD) | 4.04 (0.31) | mmol/L |
|  | Lipid panel | Cholesterol (mean +/-SD) | 174.31 (38.56) | mg/dL |
|  |  | Triglycerides (mean +/-SD) | 125.22 (77.25) | mg/dL |
|  |  | High-density lipoprotein (HDL), (mean +/-SD) | 53.33 (14.41) | mg/dL |
|  |  | Low-density lipoprotein (LDL),  (mean +/-SD) | 96.14 (31.99) | mg/dL |
|  |  | VLDL cholesterol cal (mean +/-SD) | 24.30 (13.82) | mg/dL |
|  |  | Thyroid-stimulating hormone (TSH), (mean +/-SD) | 1.93 (1.65) | uIU/mL |
|  |  | Insulin (mean +/-SD) | 13.22 (12.90) | uIU/mL |
|  | Inflammatory marker | High sensitivity C-reactive protein (CRP; hs-CRP), (mean +/-SD) | 2.29 (10.14) | mg/dL |
|  |  | White Blood Count (mean +/-SD) | 7.29 (1.96) | x10E3/μL |
|  |  | Basophil absolute (mean +/-SD) | 0.02 (0.04) | x10E3/μL |
|  |  | Basophil (mean +/-SD) | 0.45 (0.51) | % |
|  |  | Eosinophils (mean +/-SD) | 2.19 (1.61) | % |
|  |  | Eosinophils absolute (mean +/-SD) | 0.16 (0.12) | x10E3/μL |
|  |  | Lymphocyte (mean +/-SD) | 31.79 (8.31) | % |
|  |  | Lymphocyte absolute (mean +/-SD) | 2.24 (0.60) | x10E3/μL |
|  |  | Monocytes (mean +/-SD) | 7.93 (2.05) | % |
|  |  | Monocytes absolute (mean +/-SD) | 0.57 (0.19) | x10E3/μL |
|  |  | Neutrophils (mean +/-SD) | 57.63 (9.27) | % |
|  |  | Neutrophils absolute (mean +/-SD) | 4.29 (1.67) | x10E3/μL |
|  | Liver functioning tests | Aspartate aminotransferase (AST), (mean +/-SD) | 28.20 (14.08) | IU/L |
|  |  | Alanine aminotransferase (ALT), (mean +/-SD) | 29.03 (20.33) | IU/L |
|  |  | Gamma-glutamyl transferase (GGT), (mean +/-SD) | 25.73 (24.40) | IU/L |
|  |  | Alkaline phosphatase (mean +/-SD) | 72.80 (18.96) | IU/L |
|  | metabolomics | Glutamine (mean +/-SD) | 1.67E+9 (203076942) |  |
|  |  | Trans-urocanate (mean +/-SD) | 1288463.79 (769812.17) |  |
|  |  | Phenyllactate (PLA), (mean +/-SD) | 895933.22 (400227.08) |  |
|  |  | Lactate (mean +/-SD) | 316116161 (84937976.9) |  |
|  |  | Citrate (mean +/-SD) | 299922812 (42368087.1) |  |
|  |  | Pyruvate (mean +/-SD) | 18407522.7 (3764433.65) |  |
|  |  | Arginine (mean +/-SD) | 302992954 (72695839.2) |  |
|  |  | 3-hydroxyisobutyrate (mean +/-SD) | 7312525.07 (3702196.97) |  |
|  |  | 5-oxoproline (mean +/-SD) | 13280800.2 (2826601.25) |  |
|  |  | Global arginine bioavailability ratio (GABAR), (mean +/-SD) | 1.11 (0.27) |  |
|  |  | Gamma-glutamyltyrosine (mean +/-SD) | 126124.31 (54333.08) |  |
|  |  | Cortisol (mean +/-SD) | 873441.80 (377142.48) |  |
|  |  | Hypoxanthine (mean +/-SD) | 9510818.76 (7420298.18) |  |
|  |  | Decanoylcarnitine-(C10), (mean +/-SD) | 1858428.32 (2273571.08) |  |
|  |  | Hexanoylcarnitine (C6), (mean +/-SD) | 407571.41 (348790.37) |  |
|  |  | Octanoylcarnitine (C8), (mean +/-SD) | 1688905.81 (2078434.69) |  |
|  |  | Eicosanoids (mean +/-SD) | 845523.83 (391782) |  |
|  |  | Sphingosine-1-phospha(mean +/-SD)te | 2119909.39 (560524.98) |  |
|  | Methylation marks | cg11480627 (mean +/-SD) | -1.53 (0.53) |  |
|  |  | cg20704342 (mean +/-SD) | -3.37 (0.51) |  |
|  |  | cg01208318 (mean +/-SD) | -1.78 (0.69) |  |
|  |  | cg03405026 (mean +/-SD) | 5.24 (0.43) |  |
|  |  | cg03433241 (mean +/-SD) | 0.84 (0.21) |  |
|  |  | cg04112106 (mean +/-SD) | 4.21 (0.50) |  |
|  |  | cg15687973 (mean +/-SD) | -3.11 (0.41) |  |
|  |  | cg17137457 (mean +/-SD) | 2.36 (1.14) |  |
|  |  | cg20578780 (mean +/-SD) | 4.09 (0.63) |  |
|  |  | cg26454601 (mean +/-SD) | 6.26 (0.40) |  |
| **GWAS** | Polygenic risk score | PRS (mean +/-SD) | 0.0006 (0.0007) |  |

Supplementary Table 2. Sample characteristics at Phase 3 of those participants included into the analysis.

|  | | **Phase 3** | | | |
| --- | --- | --- | --- | --- | --- |
|  | **“increasing” trajectory (N=43)** | | **“resilient” trajectory (N=430)** | **Provisional PTSD (N=36)** | **No PTSD (N=437)** |
| Age | 28.26 (6.00) | | 26.75 (5.92) | 27.78 (6.17) | 26.81 (5.92) |
| Gender (%Females) | 11.6% (N=5) | | 5.3% (N=23) | 13.9% (N=5) | 5.3% (N=23) |
| PCL-5 score | 39.19 (9.81) | | 4.53 (6.04) | 41.39 (9.15) | 4.90 (6.68) |
| PHQ8 score | 11.83 (4.80) | | 2.72 (3.36) | 12.00 (4.89) | 2.85 (3.53) |
| GAD-7 score | 12.21 (4.31) | | 2.91 (3.70) | 12.53 (4.23) | 3.03 (3.84) |
| AUDIT score | 5.07 (4.07) | | 3.08 (3.05) | 5.46 (4.19) | 3.08 (3.05) |
| PSQI score | 11.71 (3.34) | | 6.42 (3.33) | 11.85 (3.63) | 6.49 (3.36) |
| DRRI-2 score | 35.74 (19.30) | | 26.95 (10.39) | 33.88 (17.47) | 27.21 (10.95) |
| TBI status: improbable | 53.5% (N=23) | | 71.6% (N=307) | 52.8% (N=19) | 71.3%(N=311) |
| TBI status: possible | 18.6% (N=8) | | 11.4% (N=49) | 19.4% (N=7) | 11.5% (N=50) |
| TBI status: mild | 20.9% (N=9) | | 14.9% (N=64) | 22.2% (N=8) | 14.9% (N=65) |
| TBI status: moderate | 7.0% (N=3) | | 1.9% (N=8) | 5.6% (N=2) | 2.1% (N=9) |
| TBI status: severe | 0% (N=0) | | 0.2% (N=1) | 0% (N=0) | 0.2% (N=1) |
| CSI current | 23.29 (11.93) | | 7.88 (9.24) | 24.50 (12.18) | 8.07 (9.27) |
| CSI lifetime | 28.39 (13.43) | | 11.57 (12.68) | 30.21 (12.61) | 11.71 (12.72) |

Note: PCL-5 = PTSD Checklist for DSM-5; PHQ8 = Patient Health Questionnaire; GAD-7 = Generalized Anxiety Disorder; AUDIT = Alcohol Use Identification Test; PSQI = Pittsburgh Sleep Quality Index; DRRI-2 = Deployment Risk and Resilience Inventory-2; TBI = Traumatic Brain Injury; CSI = Concussion Symptoms Inventory (current (past month) and lifetime (month in which symptoms were their “worst”)).

*Outcome definition: Latent Growth Mixture Modeling (LGMM)*

PTSD symptom severity trajectories through 90 to 180 days post-deployment were statistically modelled using LGMM using Mplus version 7 ^3^ and by following the statistical guidelines for LGMM ^3, 4^. To identify the best fitting number of classes a nested model approach was used, testing a progressive number of classes until the model fit indices no longer favored the addition of any more classes. Relevant criteria for determining the number of classes included the reduction in the Bayesian Information Criterion (BIC), sample-size adjusted Bayesian Information Criterion (SSBI), Akaike Information Criterion (AIC) indices, and significance indicated by the Vuong-Lo-Mendell-Rubin Likelihood (VRLT), and the Bootstrap Likelihood (BRLT), together with parsimony and interpretability. Supplementary Table 3 and 4 show the fit indices for the unconditional model (i.e. no covariates included) with all parameters free versus slope and variance fixed to 0.

Supplementary Table 3. Unconditional LGMM with all parameters free.

|  | Linear Weights Only | | | |
| --- | --- | --- | --- | --- |
| Fit indices | 1 Class^a^ | 2 Classes^a^ | 3 Classes^a^ | 4 Classes^a^ |
| AIC | 8726.977 | 8464.530 | 8273.324 | 8155.683 |
| BIC | 8760.249 | 8510.280 | 8331.551 | 8226.388 |
| SSBI | 8734.859 | 8475.368 | 8287.117 | 8172.433 |
| Entropy | – | 0.973 | 0.977 | 0.966 |
| VLRT | – | p= 0.0020 | p=0.1732 | p=0.3536 |
| LRT | – | p = 0.0027 | p=0.1822 | p=0.3634 |

Supplementary Table 4. Unconditional LGMM with variance of slope fixed to 0.

|  | Linear Weights Only | | | |
| --- | --- | --- | --- | --- |
| Fit indices | 1 Class | 2 Classes | 3 Classes | 4 Classes |
| AIC | 8781.827 | **8476.059** | 8297.187 | 8171.282 |
| BIC | 8806.782 | **8513.491** | 8347.096 | 8233.668 |
| SSBI | 8787.739 | **8484.927** | 8309.010 | 8186.061 |
| Entropy | – | **0.978** | 0.980 | 0.975 |
| VLRT | – | **p=0.0031** | p=0.1526 | p=0.1038 |
| LRT | – | **p=0.0039** | p= 0.1629 | p=0.1117 |

***Predictive modelling***

The final random forest for predicting the LGMM class memberships as outcome performed best on the training set with ten randomly sampled candidate predictor variables at each split (mtry=10), which minimized gini impurity.^5^ The final random forest for predicting provisional PTSD diagnosis using a cut-off score (PCL-5 ≥ 31) performed best on the training set using mtry=62 and “extratrees” as the splitting rule. Extremely randomized trees ("extratrees") is similar to the usual random forest algorithm but selects all splits at random instead of using Gini impurity as criterion and sometimes shows improved performance^6^. For random forest we used the C++ implementation in the R package “ranger”.^7^ Given the importance to detect “true” PTSD cases, AUC was chosen to achieve a reasonable trade-off between sensitivity, i.e., true positive rate (recall), and specificity, i.e., true negative rate, ^8^ while selecting the best tuned model exploring 100 different combinations of the hyperparameter “mtry”, “splitrule” and “min.node.size” with 1,000 trees. Synthetic minority oversampling technique (SMOTE)^9^ was used on the training set to counter high class imbalance at the data level ^9^ rather than the algorithmic level (e.g. using class weights). All results from the test dataset are reported without any prior modification of the candidate predictor data other than imputation of missing values (i.e. SMOTE is only applied during training, not testing). Little’s MCAR test showed that the missing data was likely missing completely at random (Chi-square = 6531.80, DF = 8827, p ≥.999). The removal of candidate predictors with values with near-zero variance and 45% missingness was based on pre-established theoretical consideration about missingness based on literature recommendation^10, 11^. For the evaluation metrics of the main results, 95% confidence intervals (CI) are reported. The 95% CI for the AUROC was calculated using 10,000 bootstrap resamples^12^.


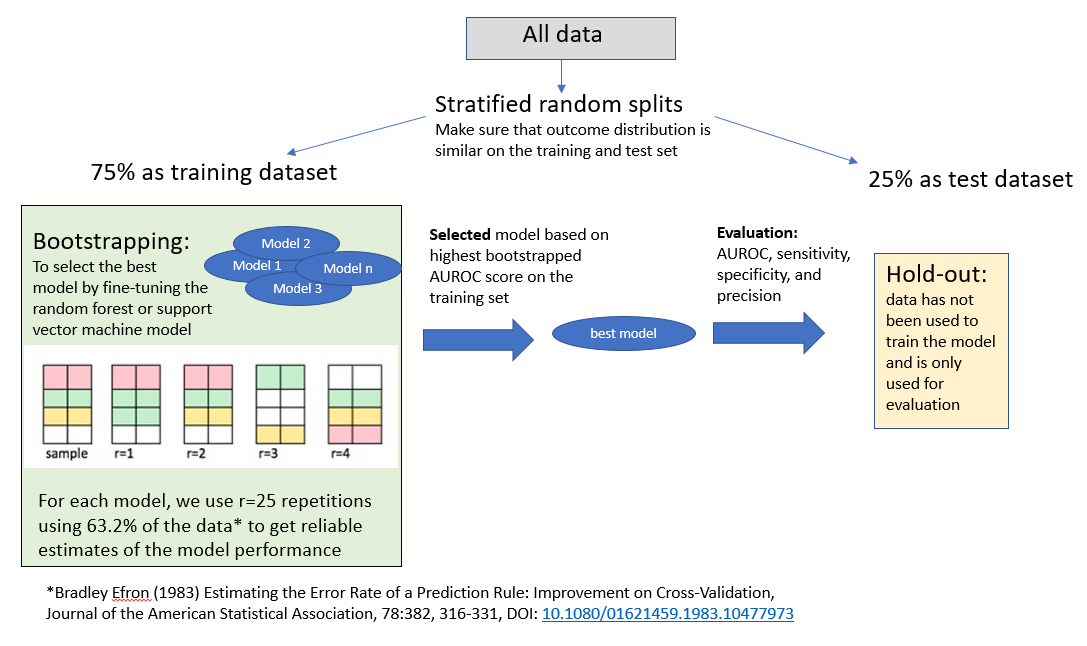


Supplementary Figure 2. Basic schematic representation of the predictive analytics approach. We use a stratified random split to generate a separate holdout test dataset to evaluate the selected “best” model from fine-tuning Random Forest (RF) and Support Vector Machine (SVM) models.

**Results**

**Model evaluation: Random Forest (RF) and Support Vector Machines (SVM):**

To evaluate the model performance in light of the class imbalance in the sample, we provide additional metrics for the presented model on the training and test set:

- **Metrics for discriminatory power,** i.e. the ability to discriminate *ex ante* between positive (“provisional PTSD diagnosis” or “increasing trajectory”) and negative events:
  - Supplementary Table 5: *Brier score*
  - Supplementary Table 6: Area Under the Receiver Operating Characteristic Curve
  - Supplementary Table 7: DeLong’s Test for correlated AUROC curves
  - Supplementary Table 11: *Cumulative gain* also known as *Accuracy Ratio*
- **Metrics for misclassification cost in imbalanced data,** i.e., metrics that account for the prevalence of PTSD (which is leading to a skewed ratio of positive and negative events) in the sample:
  - Supplementary Table 8: *Matthew’s correlation coefficient*
  - Supplementary Table 11: *Cumulative gain* also known as *Accuracy Ratio*
  - Supplementary Table 9: *Balanced accuracy*
  - Supplementary Table 10: *Precision-Recall Curve*, including the *precision*, i.e., the probability that participants with a predicted positive outcome truly have PTSD symptoms meeting the criterion of the positive outcome.

Supplementary Table 5. The Brier score is defined as the quadratic difference between the probability and the value (1,0) for our binary target classes.^13^ For comparison with the results of the main text, we add the Area under the Receiver Operating Characteristic Curve (AUROC).

|  | **Brier score** | | **AUROC (95%CI)** | |
| --- | --- | --- | --- | --- |
|  | **Trainings set:** 0.632 bootstrap estimator ^14^ | **Test set** | **Test set** | |
| Random forest: PTSD cut-off score | 0.08 | 0.11 | | 0.78 (0.67-0.89) |
| Random forest: LGMM trajectory | 0.08 | 0.11 | | 0.85 (0.75-0.96) |
| SVM: PTSD cut-off score | 0.11 | 0.10 | | 0.88 (0.78-0.98) |
| SVM: LGMM trajectory | 0.12 | 0.15 | | 0.87 (0.79-0.96) |

Since we used the AUROC to fine-tune the model parameters of the random forest models, we provide the training performance also on this metric in Supplementary Table 6.

Supplementary Table 6. Area Under the Receiver Operating Characteristic Curve (AUROC) on training and test set. The default threshold of 0.5 is used to determine sensitivity (true positive rate or recall) and specificity (true negative rate) as appropriate for the evaluation of the training performance. For the final model, the optimal threshold should be chosen to maximize clinical utility (see main text).

|  | **Trainings set:** 0.632 bootstrap estimator ^14^ | **Test set** | | |
| --- | --- | --- | --- | --- |
| Random forest: PTSD cut-off score | AUROC=**0.78** (SD=.08)  Sensitivity=0.4 (SD=.07)  Specificity=0.96 (SD=.02) | | AUROC=**0.78** [95%CI 0.67-0.89] |  |
| Random forest: LGMM trajectory | AUROC=**0.79** (SD=.07)  Sensitivity=0.43 (SD=.08)  Specificity=0.96 (SD=.03) | | AUROC=**0.85** [95%CI 0.75-0.96] |  |

To evaluate whether these predictive models are informative as indicated by the AUROC values, we can use one-sided DeLong's test for significant differences between the models of interest. First, we compare the random forest and support vector machine models with a non-informative model for each classification task (Supplementary Table 7). Second, we compare the support vector machine and random forest model among each other (Supplementary Table 7). We test the null hypothesis that the true difference between the AUC is not greater than 0 and in case of a significant p-value, we assume the alternative hypothesis that the true difference is greater than 0.

Supplementary Table 7. Shown are the test statistics for DeLong's test for two correlated ROC curves.^15^ The “non-informative model” is constructed by assigning all participants a negative outcome (majority class).

|  | **Classification tasks (test set)** | |
| --- | --- | --- |
|  | **LGMM trajectory** | **PTSD cut-off score** |
| Random forest vs.  non-informative model | Z = 6.6476, p = 1.489e-11 | Z = 4.9214, p = 4.297e-07 |
| SVM vs.  non-informative model | Z = 8.396, p < 2.2e-16 | Z=-7.3499, p = 9.915e-14 |
| SVM vs. Random forest | Z=0.403, p = 0.3435 | Z = 1.7587, p = 0.03932 |

Furthermore, the prevalence of PTSD in our study population is as low as 7.6%^16^ and in consequence the ratio of positive and negative events is imbalanced in our sample (“skewed”). To minimize skew-biased estimates of performance, the overall best evaluation metric for binary classification is said to be the Matthew’s correlation coefficient (MCC).^17^ The MCC is given by


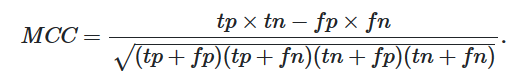


with tp: true positives; fp: false positives; tn: true negatives; fn: false negatives. The MCC ranges from -1 to 1, with 0 representing an average random prediction (MCC > 0.2 weak agreement; MCC >0.30 moderate agreement; MCC >0.70 strong agreement):

Supplementary Table 8. The Matthew’s correlation coefficient^18^. For comparison with the results of the main text, we add the Area under the Receiver Operating Characteristic Curve (AUROC).

|  | **Matthew’s Correlation Coefficient** | | | | **AUROC** |  |
| --- | --- | --- | --- | --- | --- | --- |
|  | | **Trainings set:** 0.632 bootstrap estimator^14^ | **Test set** | **Test set** | | |
| Random forest: PTSD cut-off score | | 0.744 | 0.198 | .78 | | |
| Random forest: LGMM trajectory | | 0.809 | **0.371** | .85 | | |
| SVM: PTSD cut-off score | | 0.554 | **0.308** | .88 | | |
| SVM: LGMM trajectory | | 0.504 | **0.391** | .87 | | |

These results clearly indicate that the models are not perfect but that the models perform much better than a random prediction (MCC=0) and better than models that assign all participants the majority class label (MCC=0) as well as models that assign all participants the minority class label (MCC=0).

We also provide the balanced accuracy for the training and test set in Supplementary Table 9.

Supplementary Table 9. The balanced accuracy, given by (sensitivity+specificity)/2, for applying the model (build on the training set) to the training set and to the hold-out test dataset.

|  | **Balanced Accuracy** | | | | **AUROC** | |
| --- | --- | --- | --- | --- | --- | --- |
|  | | **Trainings set:** | | **Test set** | | **Test set** |
| Random forest: PTSD cut-off score | | | 0.98 | 0.59 | | 0.78 |
| Random forest: LGMM trajectory | | | 0.97 | **0.68** | | 0.85 |
| SVM: PTSD cut-off score | | | 0.93 | **0.71** | | 0.88 |
| SVM: LGMM trajectory | | | 0.89 | **0.80** | | 0.87 |

Supplementary Table 10. Displayed is the precision and recall on the test set. Recall is the same as the sensitivity (true positive rate). Precision is defined by tp /(tp+fp). Please note that the Area Under the Precision-Recall Curve (AUPRC) “of random classifiers is 0.5 only for balanced class distributions, whereas it is P / (P + N) for the general case, including balanced and imbalanced distributions.”^19^. Table 2 of the main text shows the distribution of positive (P) events and negative (N) events in our dataset. We determine the *baseline* event rate in the entire data set by 36/ (36 + 437) = 0.076 for predicting provisional PTSD diagnosis and 43 / (43 + 430) =0.091 for predicting LGMM trajectory membership.

|  |  | **Test set evaluation** | | | | | | | |  | | | **Baseline** | | |
| --- | --- | --- | --- | --- | --- | --- | --- | --- | --- | --- | --- | --- | --- | --- | --- |
|  |  | Threshold | | | Precision | | Recall | | AUPRC | | | P/(P+N) | |  |  |
| **A** | Random forest: LGMM trajectory | | 0.523 | **0.6** | | 0.3 | | 0.41 | | | 0.09 | | | |  |
| **B** | SVM: LGMM trajectory | | 0.931 | **0.75** | | 0.3 | | 0.43 | | | 0.09 | | | |  |
| **C** | Random forest: PTSD cut-off score | | 0.554 | **0.667** | | 0.22 | | 0.29 | | | 0.08 | | | |  |
| **D** | SVM: PTSD cut-off score | | 0.945 | **0.44** | | 0.5 | | 0.30 | | | 0.08 | | | |  |


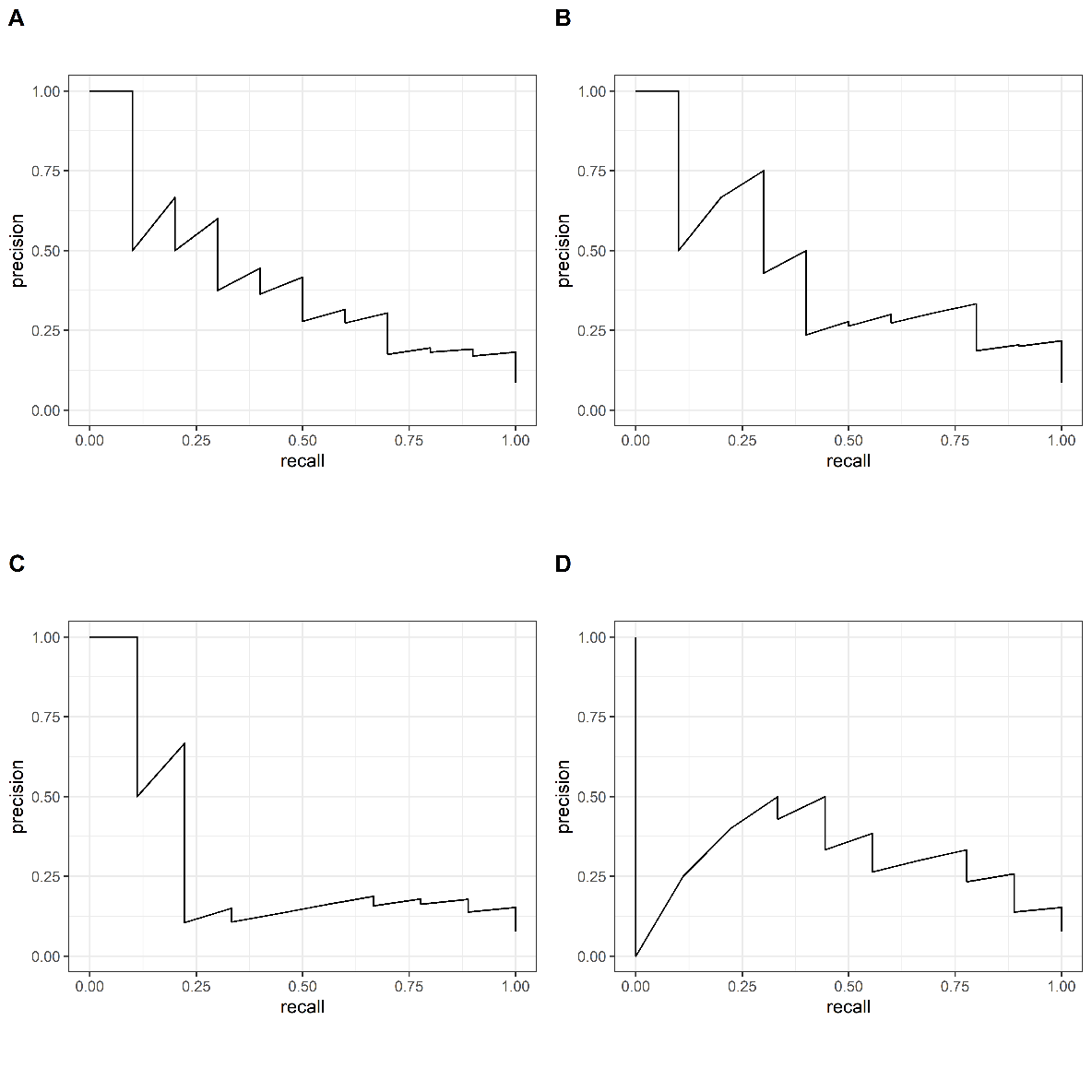


Supplementary Figure 3. Precision-Recall curve for the four different models. A Random forest: LGMM trajectory. B SVM: LGMM trajectory. C Random forest: PTSD cut-off score. D SVM: PTSD cut-off score

|  |  | **Area Under the Cumulative Gain Curve** |
| --- | --- | --- |
| **A** | Random forest: LGMM trajectory | 0.704 |
| **B** | SVM: LGMM trajectory | 0.746 |
| **C** | Random forest: PTSD cut-off score | 0.564 |
| **D** | SVM: PTSD cut-off score | 0.751 |

Supplementary Table 11 shows the cumulative gain (also known as accuracy ratio). It is calculated as the area under the black curve (Supplementary Figure 4), but above the 45 degree line, divided by the area of the gray triangle.^10, 20^ As the shape of the gray triangle depends on the distribution of positive events in the test set, this metric is useful for imbalanced samples. It indicates the direct gain of using the model compared to no using the model (45-degree line). All four models have a marked positive gain, which indicates that the models are useful.

Supplementary Figure 4. The cumulative gain curve,^10, 20^ also known as lift plot,^21^ shows the gains associated with using the model, comparing its performance with how successful we would be without the added value offered by the model. We order all the participants according to the predicted probability of the model. On the left-hand side of the x-axis are the highest probability of “provisional PTSD diagnosis” or “increasing trajectory” according to the model and the lowest probabilities for the right-hand side. In the case of A and B (classification of LGMM trajectories), we see that at the 50% point of the abscissa, we already identified 100% of the positive events, while a non-informative model would only retrieve 50% of those events. The hypotenuse of the gray triangle (the 45° reference line) represents the random model (a non-informative model), while the catheti represent the perfect model. The x-axis represents the rate of participants tested as percentage and the x-axis represents the rate of positive events (“provisional PTSD diagnosis” or “increasing trajectory”) detected.


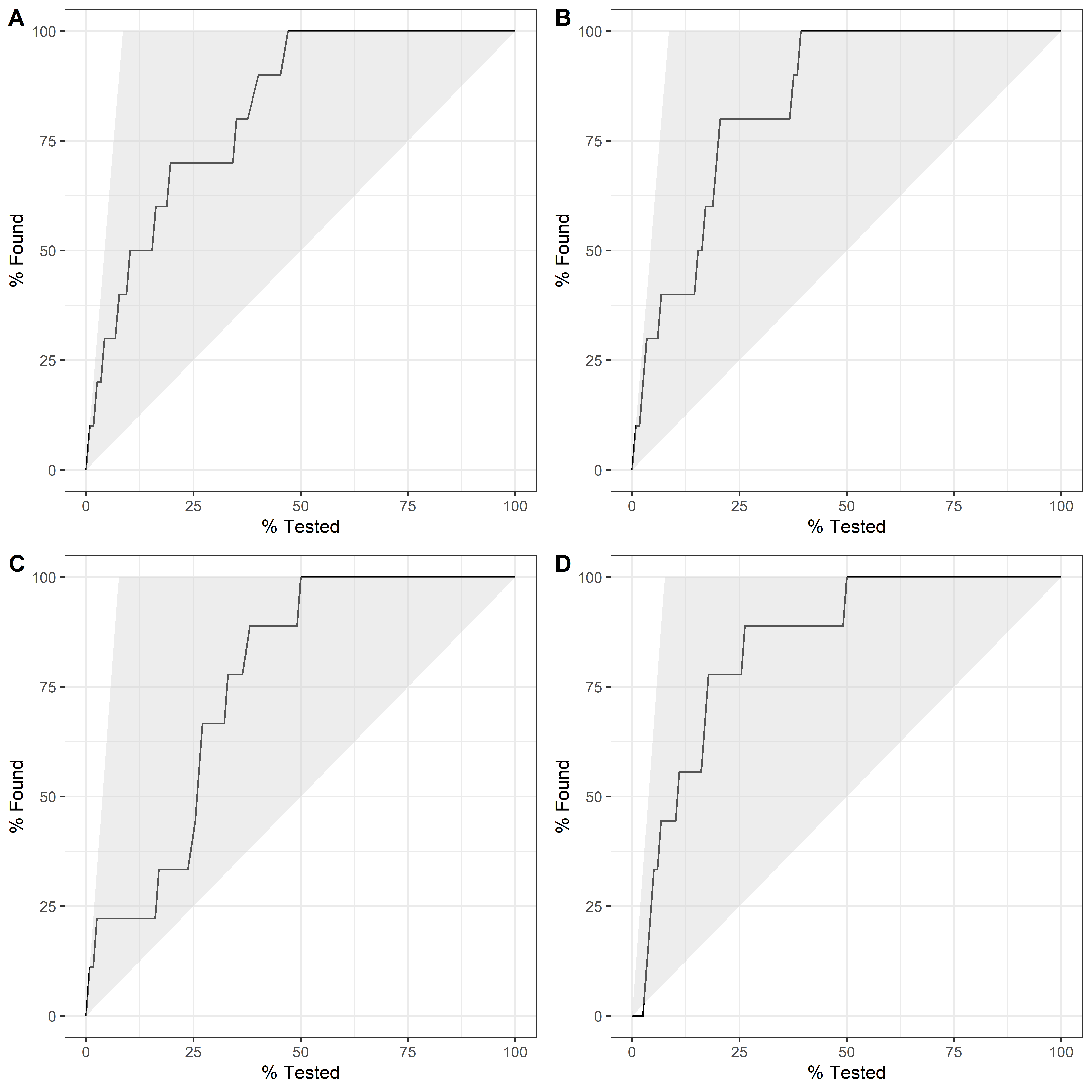


**Technical replication using a stratified 60-40 split**

In addition, we used a stratified split to partition the total dataset into a training set (60%) and test dataset (40%) for predicting provisional PTSD diagnosis. We achieved similar but slightly higher model performance (see Supplementary Figure 5 versus Figure 2 in the main text).


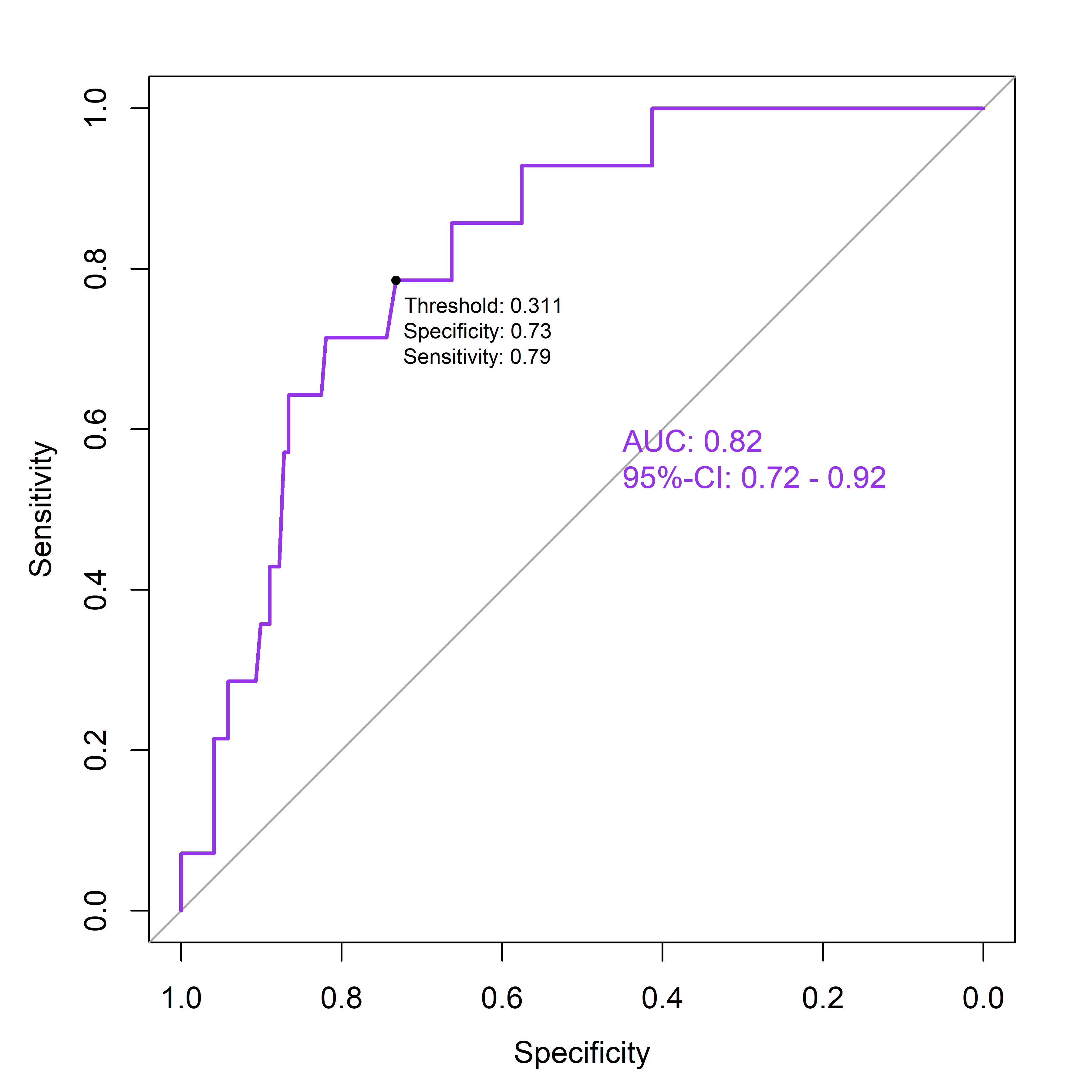


Supplementary Figure 5. Stratified split into training set (60%) and test set (40%) for predicting provisional PTSD diagnosis.


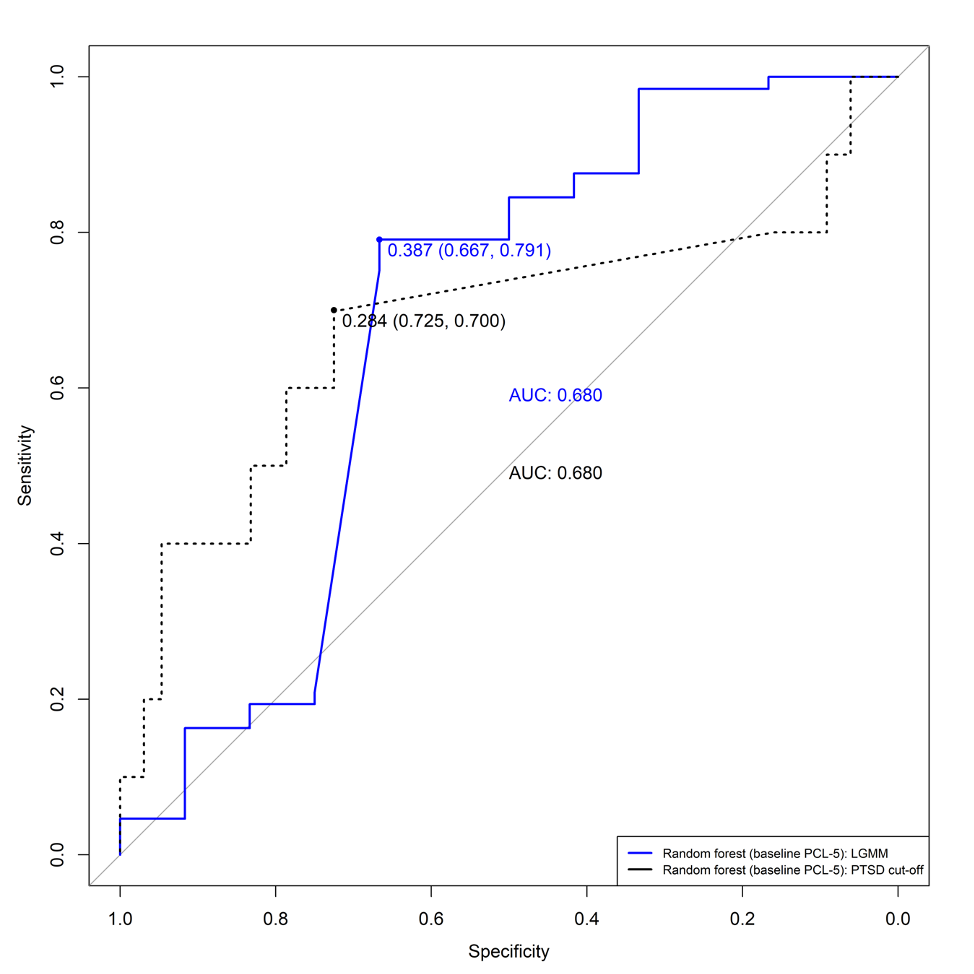


Supplementary Figure 6. Receiver Operating Characteristic Curve (ROC) for the prediction of provisional PTSD diagnosis (black dotted line; AUC = .68, with specificity = 0.73 and sensitivity =0.7) and PTSD trajectories (blue line; AUC = .68, with specificity = 0.68 and sensitivity= 0.79) only using the PCL-5 total score and its sub-items at Phase 1 (pre-deployment). Caveat: the RF model using the baseline PCL-5 score to predict the LGMM outcome is a "dummy" model and biased because the LGMM outcome implicitly statistically accounts to some degree for the baseline PCL-5 score as it models the "latent" trajectory of PCL-5 scores. Therefore, no model presented in this manuscript used any pre-deployment PTSD symptoms measured by PCL-5 as information source (except the possibly "optimistic" toy example represented by the blue line).

***Differences in warzone exposure***

We examined whether the experience of actual traumatic events during combat exposure differed between groups using section D “Combat Experiences” of the DRRI-2 assessed at Phase 2, i.e., 3 days after returning from warzone. The participants who were on the “increasing” trajectory experienced significantly more traumatic events during combat (*t*(248)=2.85, *p*=0.005; “increasing” trajectory; mean=28.89 (SD= 10.84); “resilient” trajectory; mean = 23.90 (SD=7.01)). The same was true for those participants with the provisional PTSD diagnosis according to the PCL-5 cut-off score (*t*(248)=- 3.23, *p*=0.001; provisional PTSD; mean=30.20 (SD=11.76); no PTSD; mean=23.90 (SD=6.97)).

Supplementary Table 12. To test whether there is an association between the outcome-of-interest and whether or not a variable value is missing (NA) for a participant, we calculated Pearson's χ^2^-test with Yates' continuity correction. Although the p-values seem close to a nominally significant association, the effect size indicates only a very weak association (Cramér’s V ≤ 0.1), with less clinically severe cases being more likely to have missing data.

|  | no NA | NA | Pearson's χ^2^ test with Yates' continuity correction |
| --- | --- | --- | --- |
| LGMM increasing trajectory | 22 | 21 | χ^2^= 3.4305, df=1, p = 0.064  Cramér’s V= **0.093** |
| LGMM resilient trajectory | 153 | 277 |  |
| PTSD cut-off > 31 | 19 | 17 | χ^2^=3.462, df=1, p=0.06279  Cramér’s V= **0.094** |
| PTSD cut-off < 31 | 156 | 281 |  |

***Predictor importance ranking:***

We use a permutation-based variable importance measure ^5, 22^. First, the error rate is calculated for the permuted out-of-bag (oob) data of each tree (A). Second, the total decrease in node impurities from splitting on the variable (Gini impurity) is calculated for 100 permutations (B). The results are averaged over all trees, and normalized by the standard deviation of the differences between the classification error between (A) and (B). We show importance values as scaled to 0 and 100 (Figure 3 in the main manuscript).

In addition, we show the pairwise correlation pattern between each of the top 15 predictors. Note that mutual information provided by more than one predictor may increase the uncertainty about the exact rank order of highly correlated predictors, e.g. GAD-7 and PHQ-8 are so highly correlated (Supplementary Figure 8) that we may consider them almost equally important as they are ranked in the top 5 (Figure 3 in the main manuscript).

***
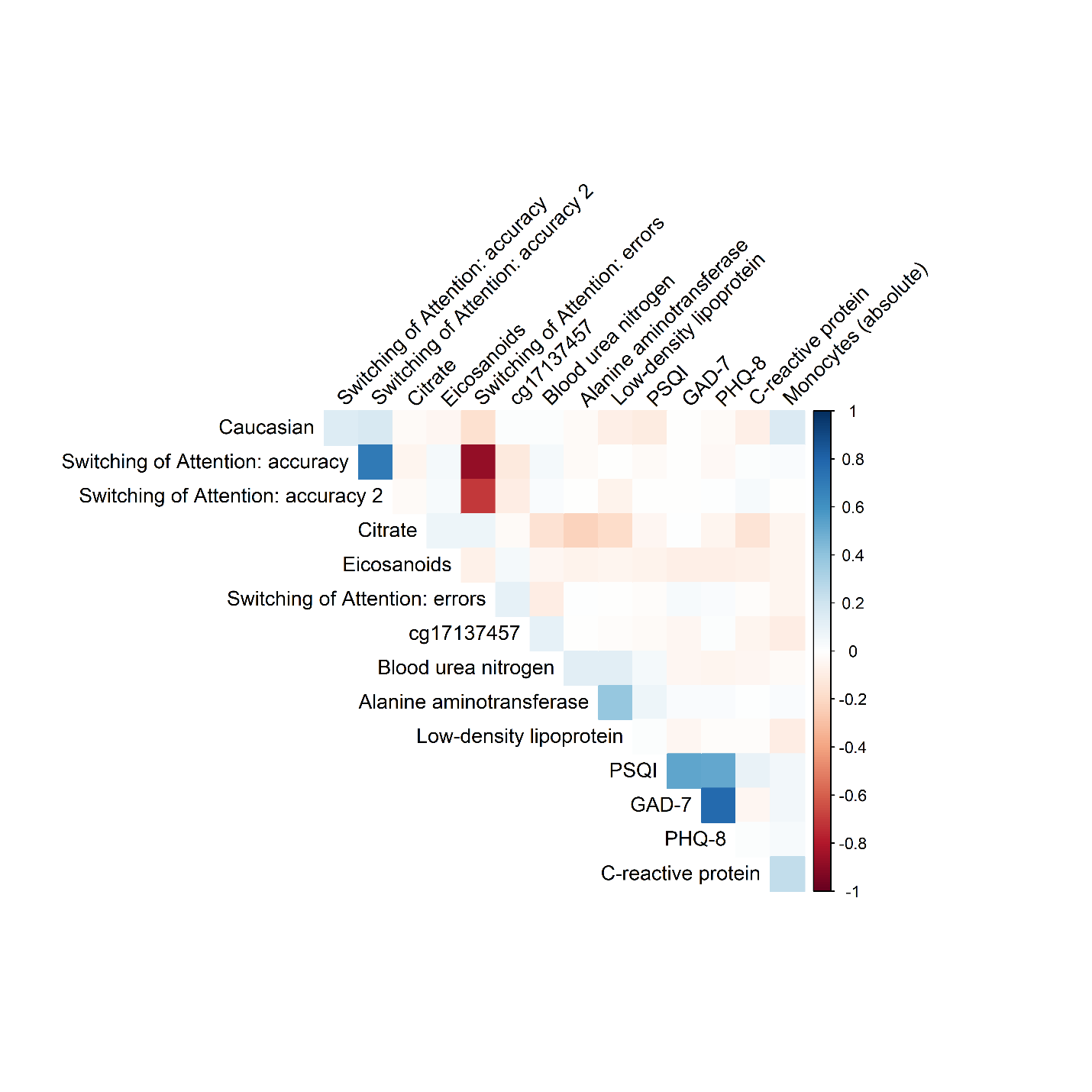
***

Supplementary Figure 7. Displayed is the correlation between the top 15 predictors of PTSD diagnosis. The mutual information of highly correlated predictors may confound the rank order of features. As shown PSQI, GAD-7, and PHQ-8 at Phase 1 are correlated. The high ranking indicates that all three are important to predict PTSD status at Phaase 3 but the correlation gives rise to uncertainty with regard to the exact ranking of those 3 predictors as the probabilistic information contributed by each is partially also provided by the others (the “mutual information”).

Supplementary Table *13.* Two Sample t-test for the most important features. There are also significant univariate mean group differences between the predicted outcome classes and the most important predictors, such as PSQI, GAD-7, PHQ-8, C-reactive protein, monocytes, glutamine (p ≤ .05) as well as exploratory trends (p ≤ .1). Results are shown for the training data as used for the variable ranking in Figure 3 of the main text.

|  | **LGMM classes of symptom trajectories** | | | **PCL cut-off score ≥ 31** | | |
| --- | --- | --- | --- | --- | --- | --- |
|  | **“increasing”** | **“resilient”** |  | **“no PTSD”** | **“provisional PTSD”** |  |
|  | **Mean** | **Mean** | **t-test** | **Mean** | **Mean** | **t-test** |
| PSQI | 7.34 | 4.92 | t(354) = 4.462, p<.001 | 5.04 | 7.78 | t(353) = -4.385, p<.001 |
| GAD-7 | 4.98 | 1.71 | t(354) = 5.836, p<.001 | 1.74 | 5.18 | t(353) = -5.785, p<.001 |
| PHQ-8 | 3.47 | 1.35 | t(354) = 4.202, p<.001 | 1.49 | 3.84 | t(353) = -3.95, p<.001 |
| cg01208318 | -1.59 | -1.82 | t(354) = 2.25, p<.05 | NA | NA | NA |
| cg17137457 | 2.14 | 2.43 | t(354) = -1.885, p<.07 | NA | NA | NA |
| Carbon Dioxide | 22.82 | 23.44 | t(354) = -1.992, p<.05 | NA | NA | NA |
| glutamine | 15.97E+8 | 16.84E+8 | t(354) = -2.927, p<.01 | NA | NA | NA |
| Citrate | NA | NA | NA | 3.02E+8 | 2.89E+8 | t(353) = 1.779, p<.08 |
| C-reactive protein | NA | NA | NA | 1.66 | 4.82 | t(353) = -3.106, p<.05 |
| Monocytes (absolute) | NA | NA | NA | 0.57 | 0.64 | t(353) = -1.993, p<.05 |

**Alternative variable importance metrics: random forest model predicting LGMM classes**

Furthermore, to evaluate the influence of individual variables on the performance of the random forest, Drop-Out Loss was explored and ^23^ with the Average Minimal Depth of a Maximal Subtree (AMDMS) metric ^24^

To evaluate the influence of individual variables on the performance of the random forest Drop-Out Loss was explored and ^23^ with the Average Minimal Depth of a Maximal Subtree (AMDMS) metric.^24^ These measures have in common that candidate predictors that are most frequently used in the earliest and largest split for building a decision tree are considered most closely related to that outcome.

**
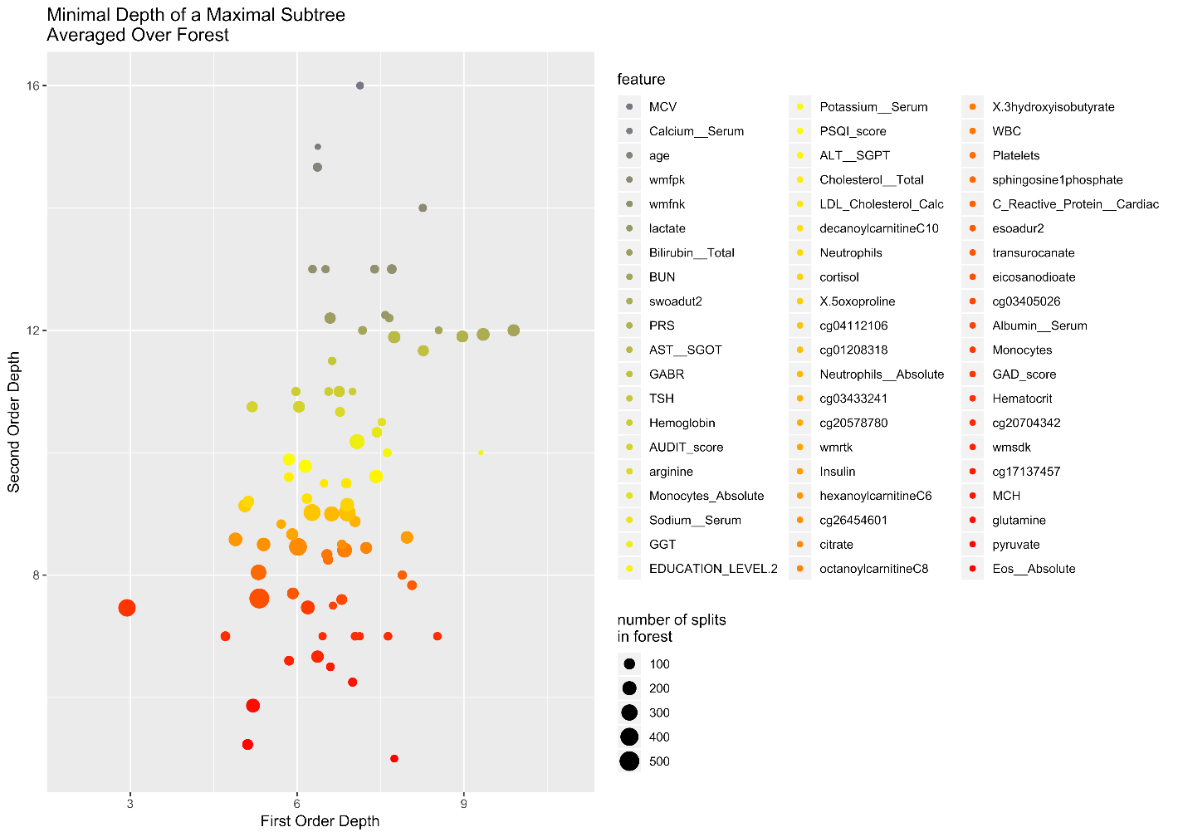
**Supplementary Figure 8. A random forest is grown out of 1,000 decision trees and final results are averaged aggregates of the classification of the individual trees.^5^ This plot shows the variable importance metric Average Minimal Depth of a Maximal Subtree (AMDMS) ^24^ for the target outcome LGMM trajectory membership. AMDMS assumes that the importance of a variable is the higher, the more frequently (dot size) the variable is used to split those nodes, where the split partitions are large number of cases of the sample (i.e. splits at minimal depth near the root of each CART). “First Order Depth” is the most minimal depth of a maximal subtree while “Second Order Depth” refers to the second most minimal depth of a maximal subtree and is used to corroborate the confidence in the first order ranking. Most important features are signified by both first and second-order AMDMS (lower left corner in red).


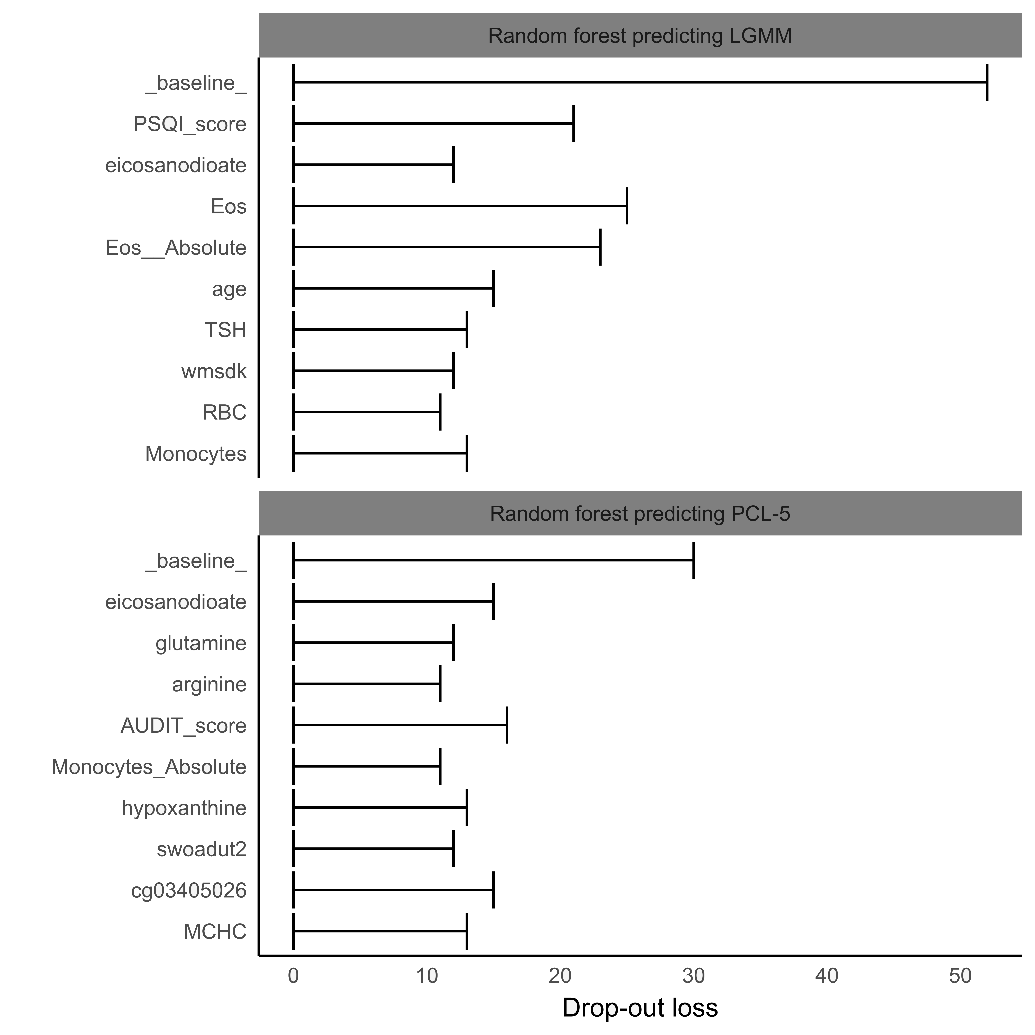


Supplementary Figure 9. Loss from Variable Dropout (Drop-out loss) is calculated by generating a sequence of different models from the random forest. The first one is the full final model. The next model is generated by shuffling the values of one particular variable so that its predictive value for the target outcome (LGMM trajectory membership or PCL-5 cut-off) is “removed” or “dropped”. By evaluating the loss of this transformation for each variable one at a time it is possible to rank the model’s variables for their predictive contribution to the random forest. The baseline drop (_baseline_) produces the biggest loss and is estimated by using the full final model but shuffling the outcome variable to introduce noise.^23^

**Variable importance for predicting PHQ-8 cut-off (depressive symptoms)**


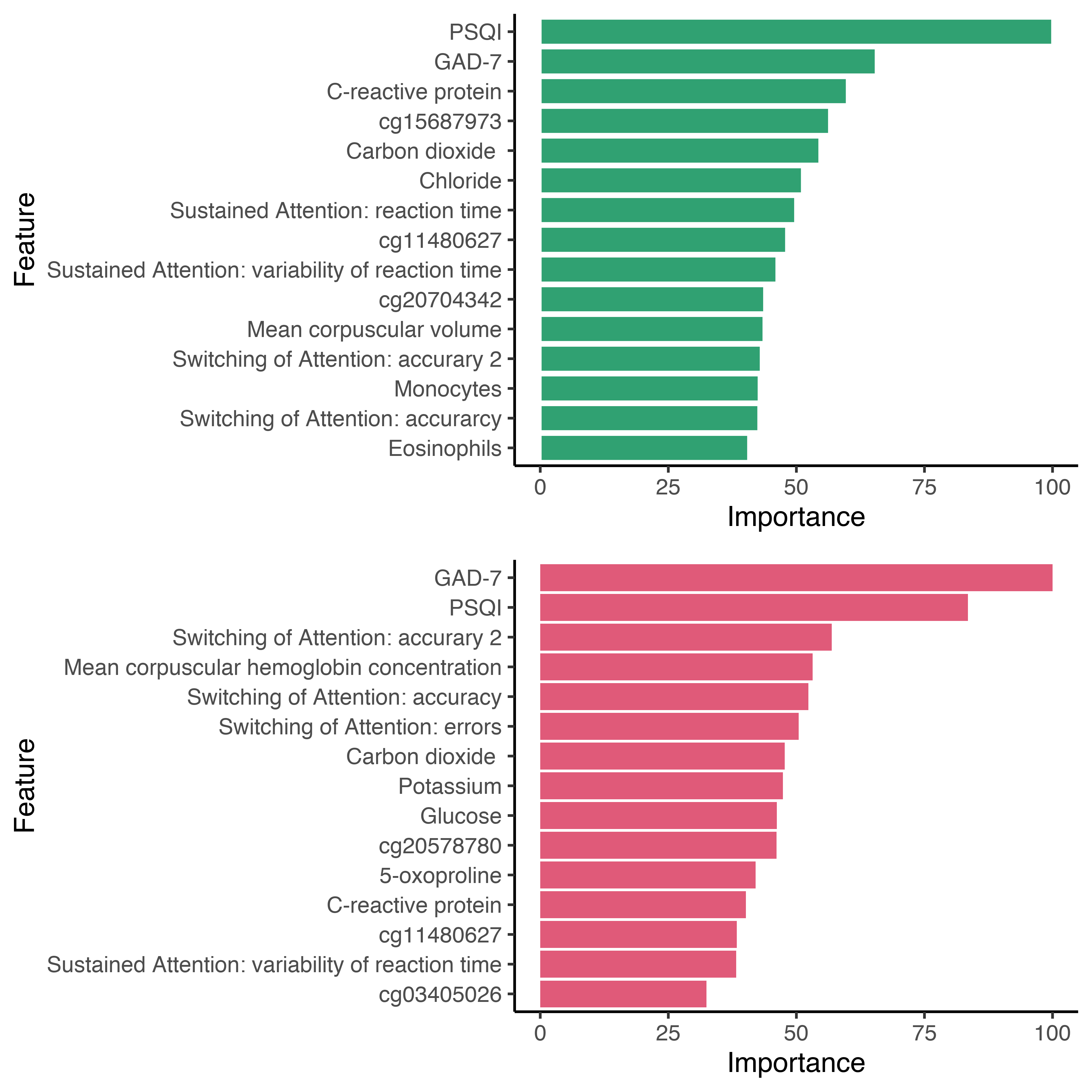


Supplementary Figure 10*.* Visualized is the variable importance for classifying those who are above the cut-off for depression according to PHQ-8 (PHQ8 ≥ 10 ^25^) (red figure; AUC = .74, sensitivity = .73, specificity = .67) and for those who are above the cut-off for depression (PHQ-8) and PTSD (PCL-5) at Phase 3 (green figure, AUC = .75, sensitivity = .83, specificity: .68).

**Variable importance for predicting provisional PTSD diagnosis using SVM**


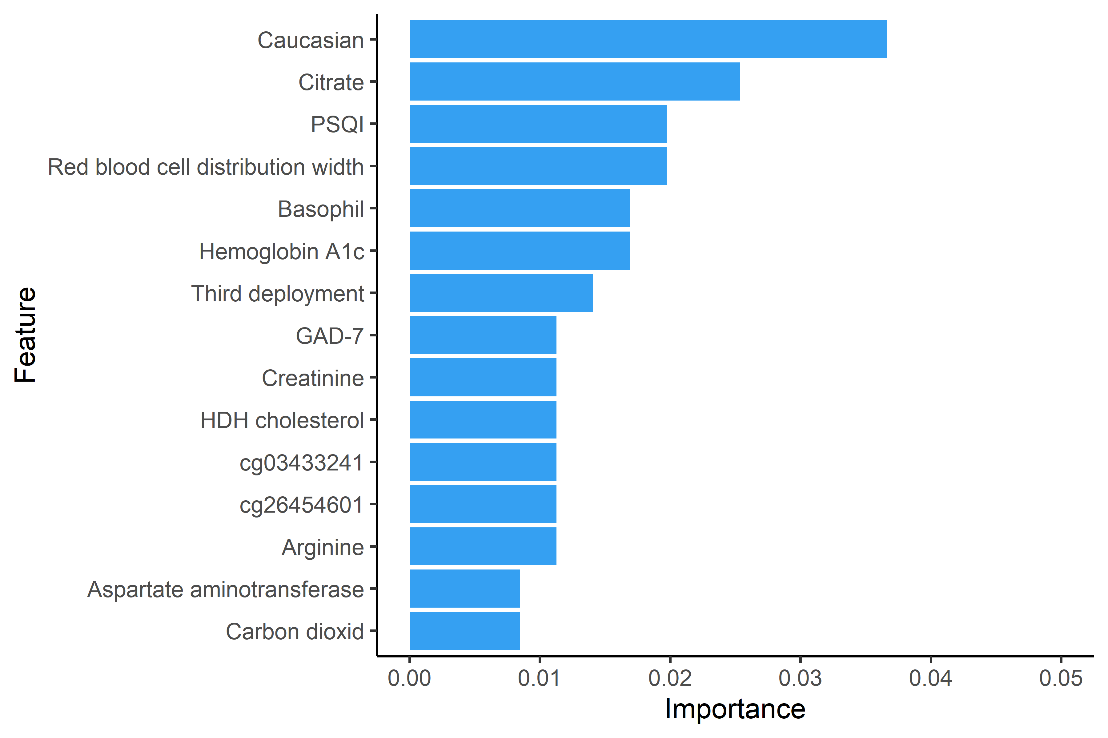


Supplementary Figure 11. Displayed are the top 15 most predictive features of the SVM model for predicting LGMM classes using 250 permutations of the outcome.

**Predicting PCL-5 scores (regression)**

We predicted PTSD symptom severity (PCL-5 scores) using random forest regression with the ranger R package using 25 times repeated bootstrapping. We obtained and Root-Mean-Squared-Error (RMSE) of 8.61, Mean Absolute Error (MAE) of 6.06 and r^2^=0.45 for predicting individual PCL-5 scores. We also predicted the probability (0,1) of being on the increasing trajectory and obtained a RMSE=0.23, MAE=0.13, and r^2^=0.43 using the random forest algorithm.

Predicting PTSD symptom severity requires larger sample than a binary classification task (Figure 2 in the main text). Using a clinical cut-off score to dichotomize the PCL-5 scores is well established for clinical decision-making^26, 27^ as clinicians ultimately need to act upon the information provided by a psychometric scale.

**Classifying LGMM trajectories using all items of the clinical self-report instruments:**

For the RF and SVM model presented in the main text, we only included the total score of all clinical self-report instruments due to potential multicollinearity among the items of those instruments. Here we use the same biomarker and neurocognitive variables but also include each of the items PHQ-8, GAD-7 and PSQI. We used regularized random forest with a penalty on introducing new predictors.


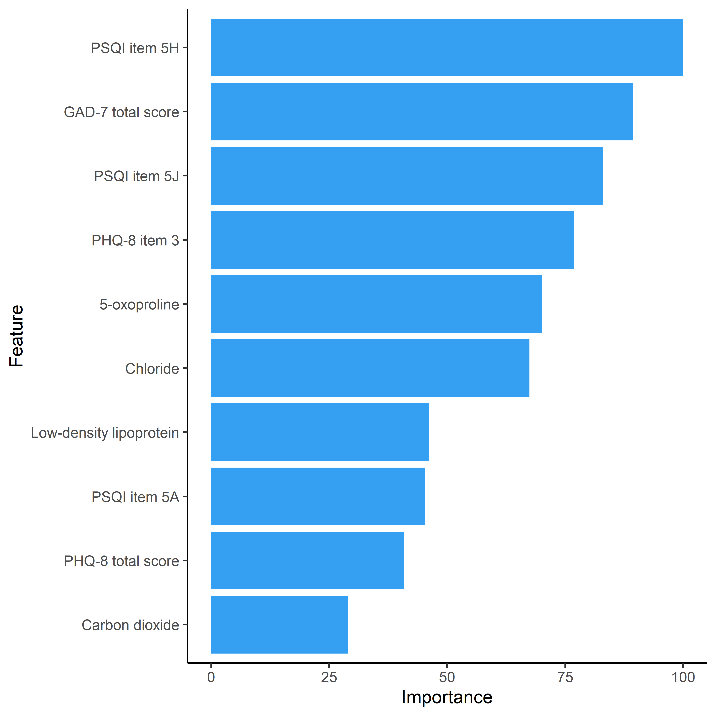


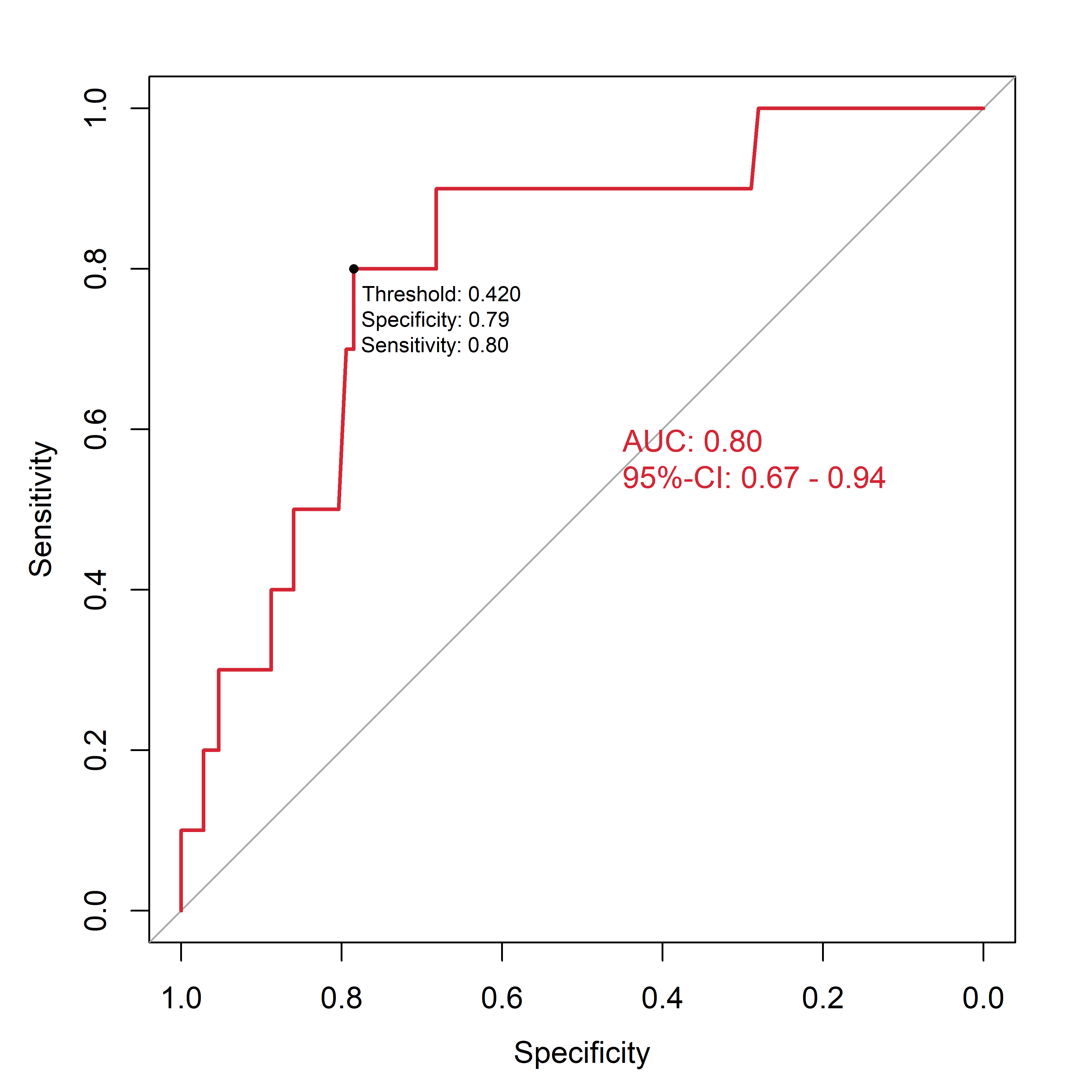


Supplementary Figure 12. Regularized random forest model using the dataset as described in the main text for predicting LGMM classes but with additional variables (all sub-items of the self-report instruments). In line with the results in Figure 2 of the main text, the total score of GAD-7 and PHQ-8 were among the most predictive features. Instead of the total score of the PSQI, the following items were important in the regularized random forest: PSQI5H (“Had bad dreams?”), PSQI5A (“Cannot get to sleep within 30 minutes?”), PSQI 5j (“How often during the past month have you had trouble sleeping because of one or more problems NOT listed above?”, and PHQ3: (“Trouble falling or staying asleep, or sleeping too much”).


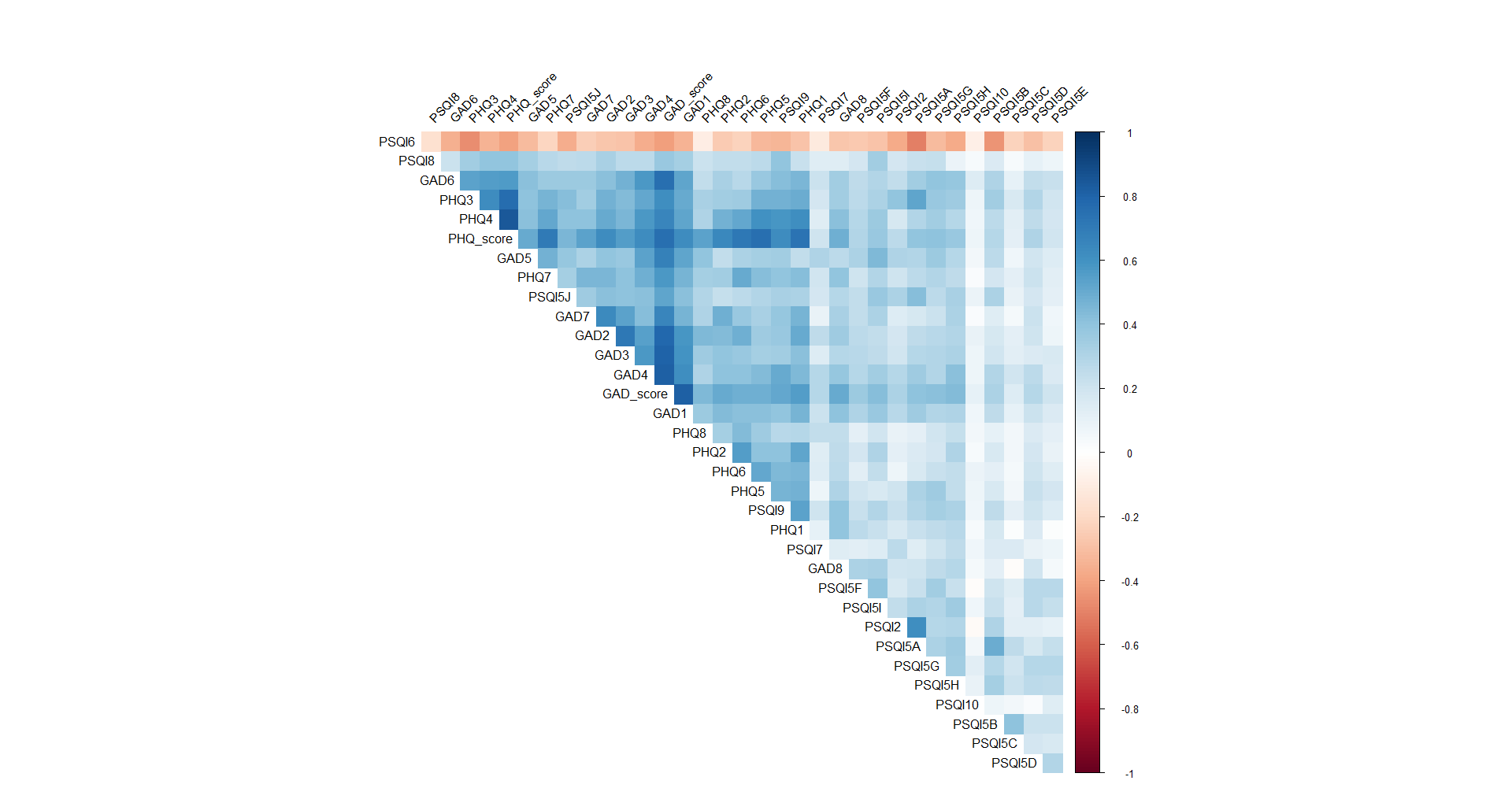


Supplementary Figure 13. Heatmap for the correlation matrix of the subitems of the psychometric instruments (PHQ-8, GAD-7, PSQI) and neurocognitive domains of WebNeuro.

Supplementary Table *14.* Shown are the test statistics for a Bootstrapped test for two ROC curves using 10,000 resamples of the data (one-sided). The null hypothesis being that the AUC of the “all feature” model (excluding the baseline PCL-5 score) is greater than the “personal baseline” model^28^ that always guesses that each individual is at a constant state, but that state can differ between individuals and is determined based on individuals pre-deployment PCL-5 score (meeting the cut-off for provisional diagnosis: yes/no).

|  | **Classification tasks (test set)** | |
| --- | --- | --- |
|  | **LGMM trajectory** | **PTSD cut-off score** |
| Random forest vs.  personal baseline model (PCL-5 baseline score) | D = 4.3691 p = 6.24e-06 | D = 3.22463 p = 0.0006 |
| SVM vs.  personal baseline model (PCL-5 baseline score) | D = 5.05339 p = 2.17e-07 | D = 4.79176 p = 8.27e-07 |

Supplementary Table 15. Null hypothesis testing with a **bootstrapped test for two ROC curves using 10,000 resamples of the data (one-sided)**: The null hypothesis being that the AUC of the “all feature” model is greater than the AUC of “PCL-5-only” model.

|  | **AUC**  Over the full range of sensitivity and specificity (0,1) including clinically non-informative portions of low sensitivity AND low specificity | |
| --- | --- | --- |
| **Model type** | **LGMM trajectory** | **PTSD cut-off score** |
| SVM using all features (excluding PCL-5)  vs.  Random forest using only the baseline PCL-5 scores | D = 1.7625, **p = 0.03899** | D = 1.6462, **p = 0.04986** |
| Random forest using all features (excluding PCL-5)  vs.  Random forest using only the baseline PCL-5 scores | D = 1.5261**,** p = 0.0635 | D = 0.83876, p = 0.2008 |

Supplementary Table 16 shows the AUC with 95% CI over the full range of sensitivity (0,1) and specificity (0,1) in the second column from the left. It also shows the partial AUC (95% CI) for a restricted range of sensitivities that are clinically most informative. The range of the partial AUC is determined by the threshold giving the greatest sensitivity for each model. The partial AUC covers the Area Under the ROC curve with sensitivity as high, or higher than the sensitivity achievable by a given model using the respective empirical best threshold of the model. The partial AUC presented in this table is standardized to the range (0,1) for the sake of interpretability as recommended in the literature ^29^. This table also presents the exact specificity and sensitivity value at the empirical best threshold for each model (see Figure 2a and 2b and Supplementary Figure 6).

| **Model type:**  *predicted outcome* | **AUC (95% CI)** | **Specificity** for empirical best threshold | **Sensitivity** for empirical best threshold | **Partial AUC (95% CI)** for thresholds of greatest **sensitivity** (above the empirical best threshold) |
| --- | --- | --- | --- | --- |
| RF using baseline PCL-5: *PTSD cut-off* | 0.68 (0.46-0.90) | 0.73 | 0.70 | 0.53 (0.44-0.84) |
| RF using all features (except  baseline PCL-5):  *PTSD cut-off* | 0.78 (0.67-0.89) | 0.71 | 0.78 | 0.78 (0.71-0.87) |
| RF using all features (except  baseline PCL-5):  LGMM trajectories | 0.85 (0.75-0.96) | 0.69 | 0.80 | 0.79 (0.73-0.92) |
| SVM using all features (except  baseline PCL-5):  *PTSD cut-off* | 0.88 (0.78-0.98) | 0.79 | 0.89 | 0.76 (0.71-0.95) |
| SVM using all features (except  baseline PCL-5):  LGMM trajectories | 0.87 (0.79-0.96) | 0.85 | 0.80 | 0.84 (0.77-0.93) |

Supplementary Table 17. shows the results of null hypothesis testing to compare the predictive models with informative benchmark models using a nonparametric **bootstrapped test (one-sided)** **for two ROC curves based on 10,000 resamples of the data.** The null hypothesis is that the partial AUC of the predictive models using “all feature” (except the PCL-5 baseline scores) are greater than the partial AUC of predictive models only using the baseline PCL-5 scores as data. The significance test is performed for the partial AUC representing only the “clinically most relevant” portions of the AUC. We define “clinically most relevant” by using the empirical best threshold that indicates an optimal trade-off between sensitivity and specificity (see Fig. 2a and 2b and Supplementary Figure 6). The partial AUC then covers only the area of the AUC where the sensitivity is as high as the sensitivity of the optimal threshold or higher.

|  | | **RF using all features** (excluding PCL-5) predicting:  **PTSD cut-off** | **RF using all features** (excluding PCL-5) predicting:  **LGMM trajectory** | **SVM using all features** (excluding PCL-5) predicting:  **PTSD cut-off** | **SVM using all features** (excluding PCL-5) predicting:  **LGMM trajectory** |
| --- | --- | --- | --- | --- | --- |
|  |  | pAUC=0.78 | pAUC=0.79 | pAUC=0.76 | pAUC=0.84 |
| **Personal baseline model**  RF using only the baseline PCL-5 score to predict post-deployment PTSD cut-off scores | pAUC=0.53 | D = 2.02971  p = 0.02119 | D = 2.11446  p = 0.01724 | D = 1.68597  p = 0.0459 | D = 1.92277  p = 0.02725 |

**References**

1. Weathers FW, Litz BT, Keane TM, Palmieri PA, Marx BP, Schnurr PP. The PTSD Checklist for DSM-5 (PCL-5). *Scale available from the National Center for PTSD at www ptsd va gov* 2013.

2. Euesden J, Lewis CM, O’reilly PF. PRSice: polygenic risk score software. *Bioinformatics* 2014; **31**(9)**:** 1466-1468.

3. Muthén LK, Muthén BO. *Mplus User’s Guide: Statistical analysis with latent variables* 8edn. Muthén & Muthén: Los Angeles, CA, 1998-2017.

4. van de Schoot R, Sijbrandij M, Winter SD, Depaoli S, Vermunt JK. The GRoLTS-Checklist: Guidelines for Reporting on Latent Trajectory Studies. *Structural Equation Modeling: A Multidisciplinary Journal* 2017; **24**(3)**:** 451-467.

5. Breiman L. Random Forests. *Machine Learning* 2001; **45**(1)**:** 5-32.

6. Geurts P, Ernst D, Wehenkel L. Extremely randomized trees. *Machine learning* 2006; **63**(1)**:** 3-42.

7. Wright MN, Ziegler A. Ranger: a fast implementation of random forests for high dimensional data in C++ and R. *arXiv preprint arXiv:150804409* 2015.

8. Fawcett T. ROC graphs: Notes and practical considerations for researchers. *Machine Learning* 2004; **31**(1)**:** 1-38.

9. Chawla NV, Bowyer KW, Hall LO, Kegelmeyer WP. SMOTE: synthetic minority over-sampling technique. *Journal of artificial intelligence research* 2002; **16:** 321-357.

10. Kuhn M, Johnson K. *Applied predictive modeling*, vol. 810. Springer2013.

11. Van Buuren S. *Flexible imputation of missing data*. Chapman and Hall/CRC2018.

12. Carpenter J, Bithell J. Bootstrap confidence intervals: when, which, what? A practical guide for medical statisticians. 2000; **19**(9)**:** 1141-1164.

13. Steyerberg EW, Vickers AJ, Cook NR, Gerds T, Gonen M, Obuchowski N *et al.* Assessing the performance of prediction models: a framework for some traditional and novel measures. *Epidemiology (Cambridge, Mass)* 2010; **21**(1)**:** 128.

14. Efron B. Estimating the error rate of a prediction rule: improvement on cross-validation. *Journal of the American statistical association* 1983; **78**(382)**:** 316-331.

15. DeLong ER, DeLong DM, Clarke-Pearson DL. Comparing the areas under two or more correlated receiver operating characteristic curves: a nonparametric approach. *Biometrics* 1988**:** 837-845.

16. Wisco BE, Marx BP, Wolf EJ, Miller MW, Southwick SM, Pietrzak RH. Posttraumatic stress disorder in the US veteran population: results from the National Health and Resilience in Veterans Study. *J Clin Psychiatry* 2014; **75**(12)**:** 1338-1346.

17. Luque A, Carrasco A, Martín A, de las Heras A. The impact of class imbalance in classification performance metrics based on the binary confusion matrix. *Pattern Recognition* 2019; **91:** 216-231.

18. Matthews BW. Comparison of the predicted and observed secondary structure of T4 phage lysozyme. *Biochimica et Biophysica Acta (BBA)-Protein Structure* 1975; **405**(2)**:** 442-451.

19. Saito T, Rehmsmeier M. The Precision-Recall Plot Is More Informative than the ROC Plot When Evaluating Binary Classifiers on Imbalanced Datasets. *PLOS ONE* 2015; **10**(3)**:** e0118432.

20. Engelmann B, Hayden E, Tasche D. Measuring the discriminative power of rating systems: Discussion Paper Series 2; 2003.

21. Data mining for direct marketing: Problems and solutions. *Proceedings of the Kdd*1998.

22. Altmann A, Tolosi L, Sander O, Lengauer T. Permutation importance: a corrected feature importance measure. *Bioinformatics* 2010; **26**(10)**:** 1340-1347.

23. Fisher A, Rudin C, Dominici FJapa. Model Class Reliance: Variable Importance Measures for any Machine Learning Model Class, from the" Rashomon" Perspective. 2018.

24. Ishwaran H, Kogalur UB, Gorodeski EZ, Minn AJ, Lauer MS. High-Dimensional Variable Selection for Survival Data. *Journal of the American Statistical Association* 2010; **105**(489)**:** 205-217.

25. Kroenke K, Strine TW, Spitzer RL, Williams JB, Berry JT, Mokdad AH. The PHQ-8 as a measure of current depression in the general population. *Journal of affective disorders* 2009; **114**(1-3)**:** 163-173.

26. Bovin MJ, Marx BP, Weathers FW, Gallagher MW, Rodriguez P, Schnurr PP *et al.* Psychometric properties of the PTSD checklist for diagnostic and statistical manual of mental disorders–fifth edition (PCL-5) in veterans. *Psychological Assessment* 2016; **28**(11)**:** 1379.

27. Bliese PD, Wright KM, Adler AB, Cabrera O, Castro CA, Hoge CW. Validating the primary care posttraumatic stress disorder screen and the posttraumatic stress disorder checklist with soldiers returning from combat. *Journal of consulting and clinical psychology* 2008; **76**(2)**:** 272.

28. DeMasi O, Kording K, Recht B. Meaningless comparisons lead to false optimism in medical machine learning. *PloS one* 2017; **12**(9).

29. McClish DK. Analyzing a portion of the ROC curve. *Medical Decision Making* 1989; **9**(3)**:** 190-195.
